# Unburdening healthcare systems through telenursing in chronic respiratory disease management: a systematic review

**DOI:** 10.3389/fdgth.2026.1746693

**Published:** 2026-05-21

**Authors:** Alessandro Vatrella, Angelo Antonio Maglio, Maria Pia Di Palo, Marina Garofano, Assunta Falco, Chiara Maria Ragusa, Vincenzo Andretta, Colomba Pessolano, Andrea Marino, Mariaconsiglia Calabrese, Alessia Bramanti

**Affiliations:** Department of Medicine, Surgery and Dentistry, University of Salerno, Baronissi, Italy

**Keywords:** asthma, chronic respiratory disease, COPD, emergency department, healthcare system, hospitalization, telemedicine, telenursing

## Abstract

**Background/objectives:**

Chronic respiratory diseases represent a major cause of morbidity/mortality and healthcare expenditure due to disease exacerbations, emergency department (ED) presentations, hospitalizations, and length of stay (LOS). This systematic review primarily aimed to evaluate the impact on the healthcare system of telenursing vs. traditional nursing care in the management of adults (≥18 years) with chronic respiratory diseases; secondarily, on treatment adherence, acceptability, and satisfaction.

**Methods:**

Electronic (PubMed, Scopus, Web of Science) and manual searches were performed until April 6, 2025, following PRISMA statement (PROSPERO CRD420251175395). Data were qualitatively synthesized; studies included were judged using dedicated tools.

**Results:**

39 studies including 4,340 patients (2,731 telenursing vs. 1,609 comparison), 3,231 with COPD and 888 with asthma, were included. Telenursing reduced exacerbation rates per patient: means 1.2 in telenursing vs. 0.9 in the comparison group at six months and 2.1 vs. 5.8 at twelve months. ED presentations remained similar (1.2 at twelve months in both groups). Hospital admissions were lower with telenursing (0.65 vs. 0.8 at six months; 0.7 vs. 1.25 at twelve months). LOS showed minimal differences (5.2 vs. 5.7 days at six months; 9.1 vs. 9.2 days at twelve months).

**Conclusions:**

Telenursing potentially slightly unburdens healthcare systems, reducing exacerbation and hospitalization rates, but did not shorten LOS once patients required inpatient care. This lack of consistency across all outcomes may suggest that telenursing may have a prevention/early detection role for clinical exacerbations, but may have limited influence on the clinical course once hospitalization becomes necessary. Further studies with standardized assessment methods are needed to evaluate treatment adherence, acceptability, and satisfaction.

**Systematic Review Registration:**

https://www.crd.york.ac.uk/PROSPERO/view/CRD420251175395, PROSPERO CRD420251175395.

## 1 Introduction

Chronic respiratory diseases are a group of diseases affecting the airways and lungs, the most common of which are chronic obstructive pulmonary disease (COPD), asthma, occupational lung disease, and pulmonary hypertension (1, 2). These chronic diseases involve millions of people worldwide every year. Several risk factors were identified, such as cigarette smoke (including passive smoking), air pollution, allergenic substances, and certain occupational activities involving contact with fine dust (2, 3).

According to the World Health Organization (WHO), the five leading respiratory diseases are not only responsible for 17% of all deaths but also contribute to 13% of healthy life years lost, either through disability, with a significant impact on patients' quality of life (QoL) (4), or premature death (2).

To effectively respond to the issues caused by chronic respiratory diseases, the WHO organized four consultative meetings, which led to the establishment, on 28 March 2006 in Beijing, China, of the Global Alliance against Chronic Respiratory Diseases (GARD), which has among its objectives the adoption of sustainable strategies and performance indicators for patient management (5).

Effective and sustainable healthcare for chronic respiratory diseases requires a comprehensive approach that integrates prevention strategies to reduce risk exposure in the general population, with optimized disease management in affected patients (2). For patients already diagnosed, early and standardized assessment, timely therapeutic intervention, and continuous monitoring are essential to limit disease progression, prevent exacerbations, emergency department presentations, hospitalization rates, and hospital length of stay (6, 7).

Furthermore, globally, chronic respiratory diseases impose a substantial burden on healthcare systems. For example, in the European Union, total annual costs for respiratory diseases exceed €380 billion, with COPD contributing approximately €38.6.4 billion (6% of total healthcare expenditures) and asthma about €33.9 billion. Meanwhile, on a global scale, the cumulative cost of COPD from 2020 to 2050 is projected to reach around Intl$4.3 trillion (data freely available on https://copd.efanet.org/cost-of-copd/#:∼:text = The%20estimated%20direct%20cost%20of%20COPD%20in%20Europe,to%20hospitalisations%2C%20followed%20by%20medication%2C%20including%20oxygen%20therapy, accessed on 04 November 2025). On a per-patient basis, asthma in Europe averages about US$1,900/year and US$3,100/year in the United States, while in low- and middle-income countries, COPD direct cost per patient may be as low as Int$52 and as high as Int$13,776 depending on disease severity and economic context.

These figures underline the urgency of implementing innovative care, such as telenursing, potentially capable of mitigating hospitalizations, reducing acute exacerbations, and thereby unburdening health services (8, 9).

In consideration of their chronic respiratory diseases' impact on the health care systems, as well as on patients' morbidity and QoL, several interventions have been developed to reduce the incidence of chronic respiratory diseases with the goal of “a world where all people breathe freely” (2).

Telenursing, or remote nursing care, is one of the modalities of eHealth and can be defined as the use of information and communication technology in the delivery of nursing services in a non-traditional manner, using information, communication, and web systems, thus enhancing a technology-based connection between the practitioner and the patient, who are physically in two different locations (10). While telenursing is part of the broader domain of telehealth and telemedicine, it is conceptually distinct in that the intervention is primarily delivered, led, or coordinated by nursing professionals and is grounded in the nursing process (assessment, planning, intervention, and evaluation). In contrast, telemedicine generally refers to physician-led remote clinical services, whereas telehealth represents a broader umbrella, including all forms of digital health delivery (10). For these reasons, in the present systematic review, telenursing was operationally defined as any telehealth intervention in which nurses had an active and central role in patient management or monitoring with chronic respiratory diseases.

The service can be provided using different types of technology, including, but not limited to, telephones, personal digital assistants, the Internet, videoconferencing and audioconferencing, computer information systems, and other modalities that allow the use of technology applied to a healthcare setting (10). However, this unconventional use does not change the traditional steps of nursing care, which still require assessment, diagnosis, planning, implementation, and evaluation of the nursing process provided, as well as the same obligations and responsibilities by the professional providing the care (11).

In the management of chronic respiratory diseases, telemedicine was largely applied for the daily patient monitoring of cardiopulmonary parameters [e.g., Forced Expiratory Volume in 1 s (FEV1), Forced Vital Capacity (FVC), and FEV1/FVC ratio], as well as to enhance treatment adherence and patients' education (7).

Early studies on the use of telemedicine (not focused on telenursing) in COPD demonstrated improvement in the QoL of patients (12). Instead, a previous study (2013), also focused only on COPD patients and highlighted the potential of telenursing programs to reduce negative clinical outcomes such as exacerbations, emergency department presentations, hospitalization rates, and length of stay (7). However, despite recent technological advancements of telemedicine programs in several fields of healthcare (13), no current research has specifically investigated the impact of telenursing on health care systems in patients with chronic respiratory diseases, including asthma, occupational lung disease, or others.

The primary objective of this systematic review is to evaluate and compare the impact on the healthcare system burden of telenursing management vs. traditional in-person nursing in the management of chronic respiratory diseases, with a focus on exacerbation rates, emergency department presentations, hospitalization rates, and length of stay. In fact, for the purpose of this systematic review, the concept of “healthcare system burden” was interpreted in terms of resource utilization, including hospital admissions, emergency department visits, and length of stay, which are commonly used indicators of healthcare system pressure. Instead, the “preventive impact” of telenursing was considered in relation to its potential to reduce exacerbations and avoid disease progression through early detection and continuous monitoring.

The secondary objective is to evaluate and compare the impact on treatment adherence, acceptability, and satisfaction of telenursing management vs. traditional in-person nursing care in patients with chronic respiratory diseases.

## 2 Materials and methods

### 2.1 Study protocol

The systematic review was conducted following the PRISMA guidelines for systematic reviews and meta-analyses (14). The protocol was defined before starting the search and was registered on the International Prospective Register of Systematic Reviews PROSPERO register with the number CRD420251175395.

The research question, structured in accordance with the PICO framework, was: “In adult patients with chronic respiratory disease, is telenursing more effective than traditional care in reducing exacerbation rates, emergency department presentations, hospitalization rates, and length of stay, with a potential unburdening on the healthcare system? And how is adherence to treatment, acceptability, and satisfaction?”

Specifically, the PICO (Participants, Interventions, Comparisons, Outcome) framework was formulated as follows (15):

- P: adult patients (age ≥ 18 years), diagnosed with a chronic respiratory disease (e.g., COPD, asthma, occupational lung disease, or pulmonary hypertension) (2);

- I: telenursing (supported by any type of technology) (10, 11, 16, 17);
- C: traditional management of chronic respiratory diseases without technology;
- O: primary outcomes: exacerbation rates, emergency department and hospital admissions rates, length of stay; secondary outcomes: treatment adherence, patient satisfaction, and acceptability.

### 2.2 Search strategy

Searches were performed in three databases: PubMed, Web of Science and Scopus, using key terms relevant to nursing and the use of tele-nursing systems in the management of chronic respiratory disease. The search strategy was initially tested in PubMed to ensure sensitivity and then adapted for use in Web of Science and Scopus, taking into account differences in database systems.

Two independent reviewers (A.F.; M.P.D.P) examined sources from the three databases. Boolean operators were used in the electronic searches and the following final same search string was used in all the databases (Supplementary Material S1 details the advanced search strategy for each database): (“Nurs*” OR “Nursing” OR “Nurse's Role” OR “nursing care” OR “Nurse-Led Interventions” OR “Nursing Practice” OR “Advanced Practice Nurse” OR “Clinical Nurse Specialist” OR “Community Health Nurse” OR “Primary Care Nurse” OR “Nursing Assessment” OR “Nursing Intervention” OR “Nursing Support” OR “Nursing Education”) AND (“Telenursing” OR “Telehealth nursing” OR “Telemonitoring” OR “Remote Monitoring” OR “Remote Patient Monitoring” OR “Telemedicine” OR “eHealth” OR “mHealth” OR “Digital Health” OR “Telecare” OR “Home Telehealth” OR “Virtual Care” OR “Health Information Technology”) AND (“Chronic Pulmonary Disease” OR “Chronic Lung Disease” OR “Chronic Respiratory Disease” OR “Chronic Obstructive Pulmonary Disease” OR “COPD” OR “Asthma” OR “Cystic Fibrosis” OR “Emphysema” OR “Pneumonia” OR “Bronchitis” OR “Pulmonary Fibrosis” OR “Interstitial Lung Disease” OR “Bronchiectasis”) AND (“Clinical Outcomes” OR “Exacerbations” OR “Hospitalizations” OR “Emergency Visits” OR “Quality of Life” OR “Lung Function Test” OR “Pulmonary Rehabilitation Outcomes” OR “Adherence” OR “Satisfaction” OR “Cost-effectiveness”).

No filters or restrictions were applied.

The electronic searches were closed on 06 April 2025.

The same two independent reviewers (A.F.; M.P.D.P.) conducted the manual search by evaluating the reference list of the included studies for potential additional records.

### 2.3 Study selection and eligibility criteria

Two independent reviewers (A.F.; M.P.D.P.), following elimination of duplicates, selected studies according to title and abstract, excluding off-topic records. The final selection to assess the inclusion or exclusion of the remaining records was conducted after full-text review. If the full text was not available, the Authors were contacted by email to obtain the manuscript. In cases of disagreement between the two independent reviewers, a third reviewer (A.B.) was involved to settle the discussion. At each stage of the study selection process, at least two independent reviewers had to agree on the final decision of each record.

The inclusion and exclusion criteria were defined to ensure clinical relevance and methodological consistency, while capturing the breadth of available evidence in the emerging field of telenursing in chronic respiratory diseases.

Inclusion criteria were identified with studies that evaluated patients aged ≥ to 18 years, with a medical diagnosis of chronic respiratory disease such as chronic obstructive pulmonary disease (COPD), asthma, occupational lung disease and pulmonary hypertension, whose management involved the use of nursing care delivered through the use of telehealth systems (supported by any type of technology) and whose outcomes included respiratory clinical parameters, quality of life, treatment adherence, patient satisfaction, exacerbations, and hospital admissions rates. Randomised clinical trials (RCTs), quasi-RCTs, non-randomised controlled clinical trials (CCTs), observational studies and case studies were included in the study to capture both experimental and real-world evidence on telenursing interventions. To ensure conceptual clarity, only studies in which nurses had an active and clearly defined role in the management or monitoring via the telehealth intervention were included. Studies describing telemedicine interventions without explicit nursing involvement were excluded.

Exclusion criteria were articles that referred to paediatric patients (age <18 years), patients without a medical diagnosis of chronic respiratory disease, or with acute respiratory disease without chronicity. Studies referring to telemedicine use without the active involvement of nurses, studies whose population consisted of patients with non-respiratory diseases or when respiratory-specific outcomes could not be clearly isolated or extracted and for whom it was not possible to extract numerical values of outcomes. Studies involving COVID-19 or respiratory diseases secondary to cancer were excluded, as their poor prognosis could confound the evaluation of telenursing effectiveness due to the acute nature of the disease, which could introduce confounding factors in the evaluation of chronic care interventions, which is the focus of the present study (17). Studies that did not report relevant clinical indicators, such as quality of life, number of exacerbations, hospitalisations or treatment adherence, were not evaluated, focusing instead exclusively on economic or technological aspects or the experiences reported by operators and/or patients, as they did not provide quantitative clinical outcomes necessary to evaluate the effectiveness of telenursing interventions. Finally, the excluded study designs were oral communication, letter to editor, case report, given their descriptive nature and they often lack rigorous methodological design or a peer-reviewed process; narrative reviews, systematic reviews, scoping reviews, umbrella reviews and study protocols, as they do not constitute primary research with extractable quantitative outcomes or may lead to duplication of evidence already captured through included primary studies.

### 2.5 Data extraction and collection

Data extraction and collection were conducted by two independent reviewers (A.F.; M.P.D.P.) and any discrepancies were resolved through discussion with a third reviewer (A.B.). Data were collected using a standard template proposed for the systematic review of interventions (18) that included selection based on the following eligibility criteria:

- Study characteristics: author, year, journal, study design, country, funding;
- Population: sample, demographic characteristics (mean/range age, gender ratio), characteristics of chronic respiratory disease (e.g., type of respiratory disease, number of previous hospitalizations, etc.), comorbidities, smoking status, ongoing pharmacological therapy.
- Interventions: type of intervention in telenursing, digital tools used, duration of intervention, timing of follow-up, other types of intervention and related duration and follow-up.
- Primary outcomes: exacerbations, emergency department presentations, hospital admissions rates, and length of stay;
- Secondary outcomes (patient-reported outcomes): treatment adherence, patient satisfaction and acceptability.

### 2.6 Data analysis

The extracted and collected data were qualitatively synthesized using Zotero and Microsoft Excel software 2019 (Microsoft Corporation, Redmond, WA, USA).

Given the clinical and methodological heterogeneity across studies, a quantitative meta-analysis was not performed. Specifically, substantial variability was observed in: (i) study designs (RCTs, non-randomized trials, observational studies); (ii) intervention characteristics (type of telenursing intervention, technological tools, intensity and duration); (iii) outcome definitions and measurement methods (e.g., non-standardized or different scales to assess the self-reported outcomes); (iv) different follow-up periods.

This heterogeneity precluded meaningful statistical pooling and raised the risk of generating misleading summary estimates.

Therefore, a structured comparative synthesis was conducted to describe and compare the characteristics and main findings of the included studies, focusing on:

1. Type of telenursing interventions (e.g., telemonitoring, tele-education, teleconsultation, mixed approaches);
2. Technological tools employed (e.g., mobile apps, telephone, web-based platforms);
3. Target populations (e.g., COPD, asthma, pulmonary fibrosis, or other chronic respiratory diseases);
4. Primary clinical outcomes (e.g., exacerbation rates, emergency department hospital presentations, length of stay);
5. Secondary patient-reported outcomes (e.g., treatment adherence, satisfaction, acceptability).

Data from each study were compared to identify common trends and differences in the effectiveness of telenursing vs. traditional management of chronic respiratory diseases by examining the direction and consistency of effects (e.g., improvement, no effect, or mixed findings). Where possible, outcome data were summarized using descriptive statistics (e.g., means, ranges), and patterns across studies were identified to highlight similarities, inconsistencies, and potential sources of variability.

Subgroup analyses were considered; however, they were not feasible due to the limited number of comparable studies within each subgroup and the inconsistency in outcome reporting.

### 2.7 Risk assessment

The studies included in this systematic review were qualitatively assessed by two independent reviewers (A.F.; M.P.D.P.) using dedicated tools for the different study designs (access performed 20 October 2025), as follows: the Risk of Bias in Nonrandomized (ROBINS-I) (freely available online on https://www.riskofbias.info/welcome/home) and the Risk of Bias for Randomized (RoB-2) (freely available online on https://www.riskofbias.info/welcome/rob-2-0-tool) studies, for non-randomized and randomized studies, respectively; the Johanna Briggs Institute (JBI) for case series tool (19).

A third reviewer (A.B.) was assigned to resolve any disagreements through discussion.

## 3 Results

### 3.1 Study selection and description

The study selection process was conducted in accordance with the Preferred Reporting Items for Systematic Reviews and Meta-Analysis (PRISMA).

The initial electronic search identified a total of 847 records as follows: PubMed returned 293 results, Web of Science returned 258 results, and Scopus returned 296 records. After removing 341 duplicate entries, 506 titles and abstracts were screened. Of these, 364 records were excluded as they were not relevant to the objectives of this systematic review.

A total of 142 full-text records were screened. Of these, 5 records, for which the full texts were not retrieved, were requested directly from the authors via email without receiving a response. Therefore, an evaluation of the remaining 137 records was conducted to evaluate their eligibility.

At the end of the study selection process, 103 records were excluded after full-text assessment due to non-compliance with the predefined eligibility criteria. The reasons for exclusion were: not telenursing intervention performed (*n* = 34); not disease-specific records (*n* = 17); review or scoping review (*n* = 12); study on technological aspects of Apps (*n* = 11); not eligible population (*n* = 10); feasibility studies of an application or the use of devices (*n* = 7); impossibility to extract data from telenursing intervention (*n* = 4); ongoing study (*n* = 4); studies only on patients' and healthcare workers' experience (*n* = 3); study protocol (*n* = 2).

The process of study selection concluded with the inclusion of 33 studies in this systematic review.

The same process was carried out for the manual search, in which the references of these 33 studies were examined. A total of 1,012 references were identified through the manual search: 247 duplicates were removed, and the remaining 765 titles and abstracts were screened, resulting in the exclusion of 746 records that were found to be irrelevant to the objectives of this systematic review.

The full texts of the 19 remaining records were examined without requiring Author contact and were evaluated for eligibility. Thirteen studies were excluded for the following reasons: not a telenursing intervention performed (*n* = 10); not disease-specific records (*n* = 2); not eligible population (*n* = 1).

The manual search led to the identification of 6 studies, eligible for inclusion in this systematic review.

Finally, based on the results of the manual and electronic searches, a total of thirty-nine (20–58) studies were included in the final analysis.

**Figure 1:**
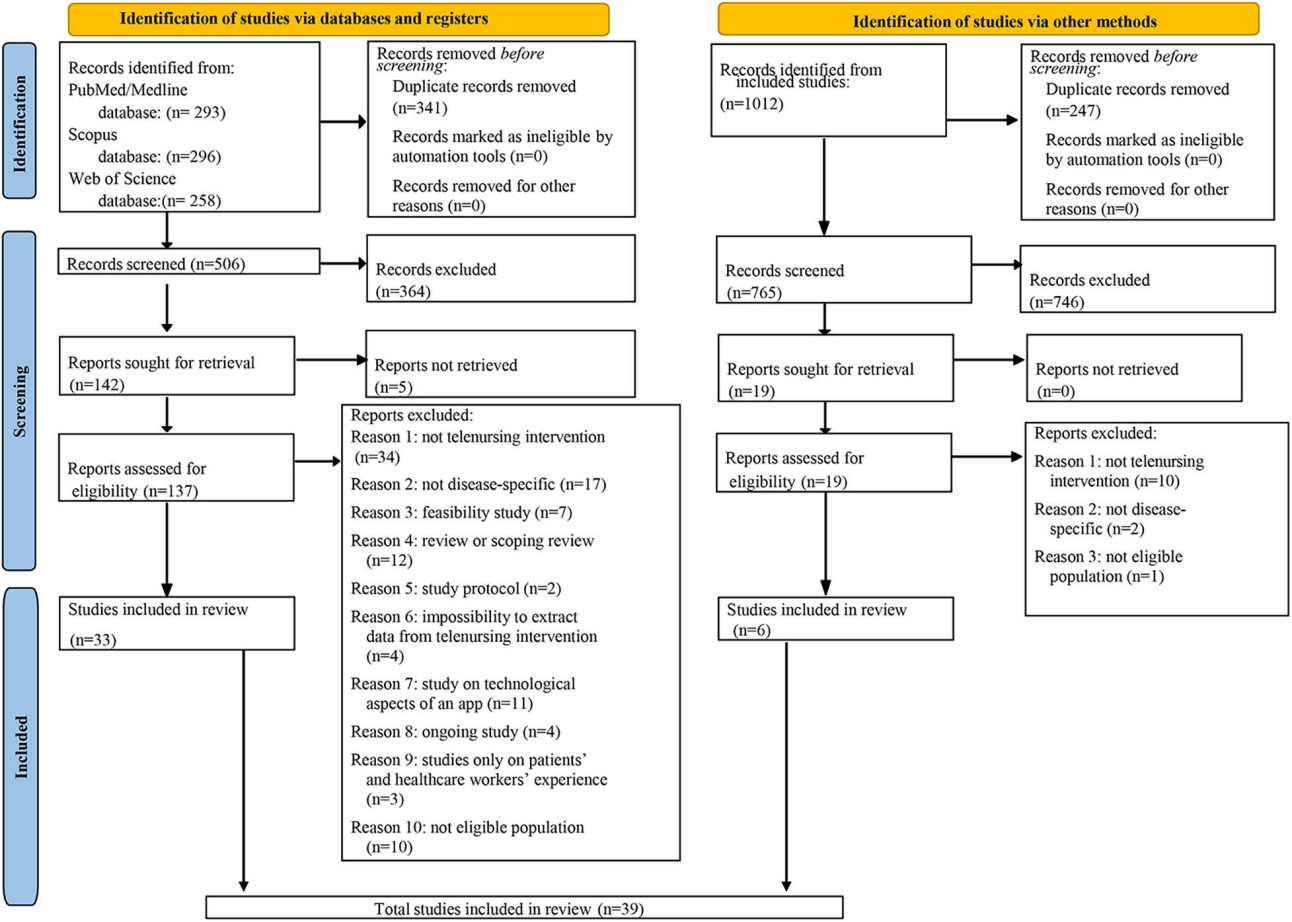
PRISMA 2020 flowchart showing the exclusion and inclusion process performed.

Figure 1 presents the PRISMA 2020 flowchart for the study selection of electronic and manual searches.

Of the 39 studies included in this systematic review, 25 were RCTs (20–24, 26, 30, 32, 33, 37–40, 42, 44, 46–49, 51–53, 55, 56, 58), 11 were prospective observational studies (25, 29, 31, 34–36, 41, 42, 45, 50, 54, 57), 1 was a retrospective observational study (41), 1 was a crossover RCT (23), and 1 was a case series (21).

Most studies were conducted in the United Kingdom (22, 23, 25, 27–29, 32, 33, 51) (*n* *=* 9), Australia (26, 43, 48, 54) and Denmark (30, 45, 52, 58) (*n* *=* 4), followed by Canada (20, 38, 53) and Netherlands (21, 46, 57) (*n* *=* 3), Japan (31, 44), United States of America (34, 35), Spain (36, 55), Norway (4, 40), Hong Kong (24, 56) (*n* *=* 2), Slovenia (37), Sweden (50), Singapore (39), and Taiwan (49) (*n* *=* 1).

The duration of the intervention performed in the study included in this systematic review varied considerably, ranging from 2 weeks (41, 55) to 26 months (54). In particular, two studies lasted 2 weeks (41, 55), one study 6 weeks (22), two studies 1 month (30, 43), one study 2 months (24), six studies 3 months (27, 29, 31, 34, 44, 56), one study 4 months (25), nine studies 6 months (20, 21, 26, 32–34, 36, 49, 57), one study lasted 6.5 months (52), one study lasted 7 months (55), one study 10 months (58), eleven studies 12 months (23, 28, 37, 42, 43, 46–48, 50, 51, 53), one study 21.5 months (38), one study lasted 24 months (39), and one study 26 months (54).

### 3.2 Telenursing

Table 1 summarizes the studies and population characteristics, the characteristics of interventions performed in telenursing, and the related patients' reported outcomes (intervention group).

**Table 1:** Telenursing (intervention) and traditional care (comparison) groups: studies and population characteristics, the characteristics of interventions performed with and without telenursing, and the related patients’ reported outcomes.

Study	Population	Intervention	Patient-reported Outcomes
Ahmed et al., 2017 (20) *JMIR* Pilot RCT Canada *Canadian Institutes of Health Research operating grant f*unded	**Sample size**: 47 **Mean age/age range:** MD/18-69 **Gender ratio (M/F):** 15M/32F **Pulmonary disease**: Asthma ED visits due to a respiratory problem (in the previous 3 months): 0 (n.37), 1(n.6), ≥ 2 (n.2) Number of hospitalizations due to respiratory issues (in the previous 3 months): 0 (n.44), 1 (n.1), ≥ 2 (n.0), missing (n.2) **Comorbidities:** N/D **Smoking:** n.5 (current smoker), n.15 (former smoker) **Ongoing pharmacological therapy**: N/D asthma treatment drugs	**Type of intervention in telenursing:** real-time monitoring and support from a nursing case manager for discussing medications, clarifying the action plan, and conducting follow-ups related to control status **Digital tool used:** emails, phone and a specific portal website **Duration:** 6 months **Follow-up**: 3, 6 and 9 months **Other type of intervention**: N/D **Duration**: N/D **Follow-up**: N/D	**Adherence**: N/D **Satisfaction**: N/D **Acceptability:** N/D
	**Sample size**: 51 **Mean age/age range:** MD/18–69 **Gender ratio (M/F):** 18M/33F **Pulmonary disease**: Asthma diagnosis ED visits due to a respiratory problem: 0 (n.38), 1(n.3), ≥ 2 (n.4), missing (n.6) Number of hospitalizations due to respiratory issues: 0 (n.43), 1 (n.1), ≥ 2 (n.1), missing (n.6) **Comorbidities:** N/D **Smoking:** n.7 (current smoker), n.16 (former smoker), n.2 (missing) **Ongoing pharmacological therapy**: unspecified asthma treatment drugs	**Type of intervention:** Standard care **Duration:** 6 months **Follow-up**: 3 and 6 months	**Adherence**: N/D **Satisfaction**: N/D **Acceptability:** N/D
Antoniades et al., 2012 (48) *Telemedicine and e-health* Pilot study Australia No funded	**Sample size**: 22 **Mean age/age range:** 70 **Gender ratio (M/F):** 10M/12F **Pulmonary disease**: diagnosis of COPD (moderate to severe) COPD-related admissions (in the previous 12 months), median: n. 2 COPD-related LOS (in the previous 12 months), median: n. 16 **Comorbidities:** MD **Smoking:** n.0 (current smoker), n.4 (nonsmoker) **Ongoing pharmacological therapy**: MD	**Type of intervention in telenursing:** Daily home remote monitoring of symptoms, medication use and vital signs (spirometry, weight, temperature, blood pressure, oxygen saturation, electrocardiogram, sputum color and volume) **Digital tool used:** laptop with simplified interface and telemonitoring devices (digital blood pressure cuff, digital stethoscope, pulse oximeter, thermometers, scales, electrocardiogram touch plate and pneumotachograph) **Duration:** 12 months **Follow-up**: 6 and 12 months **Other type of intervention**: best practice care **Duration**: 12 months **Follow-up**: N/D	**Adherence**: excellent at a median of 80% to daily measurements (adherence for specific parameters: SpO_2_:81%; blood pressure: 83%; weight: 80%; relaxed spirometry: 40%; forced spirometry: 79%; body temperature:81%; questionnaires:73%) **Satisfaction**: 88% overall satisfaction rate (94% of patients describing the telemonitoring system as easy to use; 82% feeling the system helped them manage their COPD better) **Acceptability:** high, although more than half of the patients had never used a computer, the majority adapted well to the technology, considering it useful and non-invasive
	**Sample size**: 22 **Mean age/age range:** 68 **Gender ratio (M/F):** 10M/12F **Pulmonary disease**: diagnosis of COPD (moderate to severe) Hospital admissions (in the previous 12 months), median: n.1 LOS (in the previous 12 months), median: n.8 **Comorbidities:** N/D **Smoking:** n.6 (current smoker), n.2 (nonsmoker) **Ongoing pharmacological therapy**: N/D	**Type of intervention:** best practice care **Duration:** 12 months **Follow-up**: 6 and 12 months	**Adherence**: N/D **Satisfaction**: N/D **Acceptability:** N/D
Berkhof et al., 2015 (21) *ASPR* Pilot study Netherlands No funded	**Sample size**: 52 **Mean age/age range:** 68 ± 9 **Gender Ratio (M/F**): 34M/18F **Pulmonary disease**: COPD diagnosis Number of hospitalizations (in past 12 months): n.23 **Comorbidities:** MD **Smoking:** MD **Ongoing pharmacological therapy**: HOT: n.9; inhalation medication: n.30 (short-acting), n.48 (long-acting), n.49 (corticosteroids)	**Type of intervention in telenursing:** structured phone calls every 2 weeks during which the Clinica COPD Questionnaire was conducted **Digital tool used:** phone **Duration**: 6 months **Follow-up**: 6 months **Other type of intervention**: N/D **Duration**: N/D **Follow-up**: N/D	**Adherence**: N/D **Satisfaction**: N/D **Acceptability:** N/D
	**Sample size**: 49 **Mean age/age range:** 68 ± 9 **Gender Ratio (M/F**): 34M/15F **Pulmonary disease**: COPD diagnosis Number of hospitalizations (in past 12 months): n.17 **Comorbidities:** N/D **Smoking:** N/D **Ongoing pharmacological therapy**: HOT: n.6; inhalation medication: n.32 (short-acting), n.39 (long-acting), n.45 (corticosteroids)	**Type of intervention:** regular outpatient visit at baseline and after 6 months by the pulmonologist. Interim outpatient visits were planned at 2 and 4 months with a pulmonary nurse practitioner. **Duration:** 6 months **Follow-up**: 6 months	**Adherence**: N/D **Satisfaction**: N/D **Acceptability:** N/D
Billington et al., 2014 (22) *JCOPD* RCT United Kingdom No funded	**Sample size**: 35 **Mean age/age range:** 72.09 **Gender Ratio (M/F**): 18M/17F **Pulmonary disease**: COPD diagnosis (GOLD grading n.12 (mild), n.23 (moderate) **Comorbidities:** MD **Smoking:** n.20 (current smoker), n.10 (former smoker), n.5 (never smoker) **Ongoing pharmacological therapy**: MD	**Type of intervention in telenursing:** COPD self-management plan with nurse telephone support **Digital tool used:** telephone **Duration:** 6 weeks **Follow-up**: 12 weeks **Other type of intervention**: N/D **Duration**: N/D **Follow-up**: N/D	**Adherence**: N/D **Satisfaction**: assessed through 6 single-choice questions not part of a validated scale. Improved across all items from baseline to follow-up **Acceptability:** monitored by looking at both participation and attrition rate but not quantified
	**Sample size**: 38 **Mean age/age range:** 71.97 **Gender ratio (M/F):** 17M/21F **Pulmonary disease**: COPD diagnosis (GOLD grading n.18 (mild), n.20 (moderate) **Comorbidities:** N/D **Smoking:** n.19 (current smoker), n.11 (former smoker), n.8 (never smoker) **Ongoing pharmacological therapy**: N/D	**Type of intervention:** Self-management plan alone on patient well-being and symptom severity **Duration:** 6 weeks **Follow-up**: 12 weeks	**Adherence**: N/D **Satisfaction**: N/D **Acceptability:** N/D
Chatwin et al., 2016 (23) *BMJ* Crossover RCT United Kingdom *National Institute for Health Research* funded	**Sample size**: 72 **Mean age/age range:** 61.8 **Gender Ratio (M/F):** 29M/43F **Pulmonary disease**: COPD (n.39) and chronic respiratory failure (n.33) **Comorbidities:** heart failure (n.9), diabetes (n.14), chronic renal failure (n.0), hypertension (n.26), cerebral vascular accident/transient ischaemic attack (n.1), myocardial infarction (n.1), angina (n.1), pulmonary hypertension (n.4) **Smoking:** N/D **Ongoing pharmacological therapy**: NIV: 52; LTOT: 38	**Type of intervention in telenursing (group1: n.38 patients/n.19 with COPD):** telemonitoring for the first 6 months. The data was analysed by hospital nurses, who intervened based on traffic light alerts, providing clinical indications or involving other professionals **Type of intervention in telenursing (group 2: n.34 patients/20 COPD):** standard best practice care (6 months) and delayed telemonitoring (next 6 months) **Digital tool used:** Telemonitoring devices (oximetry, heart rate, blood pressure and weight) and phone **Duration:** 12 months **Follow-up**: 6 and 12 months **Other type of intervention**: N/D **Duration**: N/D **Follow-up**: N/D	**Adherence**: N/D **Satisfaction**: N/D **Acceptability:** N/D
Cooper et al., 2009 (25) *Journal of Assistive Technologies* Prospective observational study United Kingdom No Funded	**Sample size**: 10 **Mean age/age range:** 70 **Gender ratio (M/F):** MD **Pulmonary disease**: diagnosis of COPD: n.5 (mild), n.2 (moderate), n.3 (severe) **Comorbidities:** MD **Smoking:** MD **Ongoing pharmacological therapy**: MD	**Type of intervention in telenursing:** the respiratory nurse was responsible for data monitoring, triage calls, home visits in case of emergency, and education on the use of devices and the management of exacerbations. The nurse also provided a “patient advice card” with instructions on what to do based on vital signs results. **Digital tool used:** a computer and telemonitoring devices (spirometer capable of measuring both peak flow and FEV1, and pulse oximeter, which measured both pulse rate and the saturated level of blood oxygen) **Duration:** 4 months **Follow-up**: daily **Other type of intervention**: N/D **Duration**: N/D **Follow-up**: N/D	**Adherence**: N/D **Satisfaction**: Evaluated using an unspecified method. However, all respondents stated that they felt the device had become a useful part of their care by the end of the pilot project. The telemedicine group found the measurement regimen to be somewhat inconvenient but was still deemed positive. **Acceptability:** N/D
	**Sample size**: 9 **Mean age/age range:** 78 **Gender ratio (M/F):** MD **Pulmonary disease**: diagnosis of COPD: n.3 (mild), n.3 (moderate), n.3 (severe) **Comorbidities:** MD **Smoking:** MD **Ongoing pharmacological therapy**: MD	**Type of intervention in telenursing:** telemedicine system with a vital signs instrument, biweekly telephone contact by a respiratory nurse who manually recorded data and provided counseling as needed. **Digital tool used:** a system using standalone devices with manual data collection over the telephone **Duration:** 4 months **Follow-up**: biweekly **Other type of intervention**: N/D **Duration**: N/D **Follow-up**: N/D	**Adherence**: N/D **Satisfaction**: N/D method. However, all respondents stated that they felt the device had become a useful part of their care by the end of the pilot project (the standalone group was entirely happy with the level of intervention and monitoring). **Acceptability:** N/D
De San Miguel et al., 2013 (26) *Telemed JE Health* RCT Australia Funded by *Australian Department of Health and Ageing*	**Sample size**: 36 **Mean age/age range:** 71 **Gender Ratio (M/F**): 14M/22F **Pulmonary disease**: COPD diagnosis **Comorbidities:** MD **Smoking:** MD **Ongoing pharmacological therapy**: MD	**Type of intervention in telenursing:** daily, a nurse monitored the data that was transmitted by the patients and called them to discuss the results, provide advice **Digital tool used: a** small portable unit, telemonitoring devices (blood pressure, weight, temperature, pulse, oxygen saturation level) and phone **Duration:** 6 months **Follow-up**: **daily** **Other type of intervention**: N/D **Duration**: N/D **Follow-up**: N/D	**Adherence**: N/D **Satisfaction**: detected via face-to-face interview, but not quantifiable. Participants described having more control over their condition and being more confident in self-managing their condition, as they were now more conscious of what their body was doing. **Acceptability:** N/D
	**Sample size**: 35 **Mean age/age range:** 74 **Gender Ratio (M/F**): 20M/15F **Pulmonary disease**: COPD diagnosis **Comorbidities:** N/D **Smoking:** N/D **Ongoing pharmacological therapy**: N/D	**Type of intervention:** information for disease management only **Duration**: 6 months **Follow-up**: 6 months	**Adherence**: N/D **Satisfaction**: N/D **Acceptability:** N/D
Dinesen, 2012 (58) J Telemed Telecare Denmark RCT No funding	**Sample size**: 57 **Mean age/age range**: 68 **Gender ratio (M/F):** MD **Pulmonary disease:** COPD (stage III–IV) COPD hospitalizations (in the last year): MD **Comorbidities:** MD **Smoking:** MD **Ongoing pharmacological therapy**: MD	**Type of intervention in telenursing**: Home tele-rehabilitation **Digital tool used:** Telemonitoring devices (spirometer, pulse-oximeter, heart rate monitor, balance) + call center **Duration:** 10 months **Follow-up:** 10 months **Other type of intervention**: physical exercises (sitting exercises on a chair, stretching of neck muscles, exercises for the legs, standing exercises for arms and chest cavity, walking exercises) **Duration**: 4 months **Follow-up**: 10 months	**Adherence**: N/D **Satisfaction**: N/D (the patients reported that the technology helped them to visualize the data and obtain an overview of the development of symptoms) **Acceptability:** N/D
	**Sample size:** 48 **Mean age/age range:** 68 **Gender ratio (M/F):** N/D **Pulmonary disease:** COPD (stage III–IV) COPD hospitalizations in the last year: N/D **Comorbidities:** N/D **Smoking:** N/D **Ongoing pharmacological therapy:** N/D	**Type of intervention:** Standard care (exercise education, no active monitoring or support) **Duration:** 10 months **Follow-up:** 10 months	**Adherence**: N/D **Satisfaction**: N/D **Acceptability:** N/D
Early et al., 2017 (27) *International J COPD* Case series United Kingdom *NHS East of England Regional Innovation Funding* funded	**Sample size**: 11 (group 1), 8 (group 2) **Mean age/age range:** 66.36 (group 1), 60.63 (group 2) **Gender ratio (M/F):** 6M/5F (group 1), 3M/5F (group 2) **Pulmonary disease**: COPD according to GOLD stage, group 1: III-IV, group 2: II-III COPD exacerbation (in the last year), mean: 3.7 (group 1), 7(group 2) Hospital admission in past year, median: 4(group 1), 0 (group 2) **Comorbidities:** N/D **Smoking:** current smoker n. 3 (group 1), n.5 (group 2) **Ongoing pharmacological therapy**: O_2_ therapy: n.5 (group 1), n. 0 (group 2)	**Type of intervention in telenursing:** online resource with integrated nursing coaching for self-management **Digital tool used:** email and phone **Duration:** 3 months **Follow-up**: 3 months **Other type of intervention**: N/D **Duration**: N/D **Follow-up**: N/D	**Adherence**: N/D **Satisfaction**: N/D **Acceptability:** N/D
Farmer et al., 2017 (28) *J Medical Internet Research* RCT United Kingdom No funded	**Sample size**: 110 **Mean age/age range:** 69.8 **Gender ratio (M/F):** 68M/42F **Pulmonary disease**: COPD (GOLD score moderate (n.41), severe or very severe (n.69) Number of COPD medications, median: 5 Number of other medications, median: 4 **Comorbidities:** high blood pressure, osteoporosis, high cholesterol, diabetes, heart disease, depression: n.89 **Smoking:** 23 (current smoker), n. 87 (former smoker) **Ongoing pharmacological therapy**: N/D	**Type of intervention in telenursing:** telemonitoring and self-management support **Digital tool used:** tablet and Bluetooth oximeter **Duration:** 12 months **Follow-up**: 3,6 and 12 months **Other type of intervention**: home visits **Duration**: 12 months **Follow-up**: 12 months	**Adherence**: rated through MARS: from 23.4 (at baseline) to + 0.17 (at 12 months) **Satisfaction**: N/D **Acceptability:** N/D
	**Sample size**: 56 **Mean age/age range:** 69.8 **Gender ratio (M/F):** 34M/22F **Pulmonary disease**: COPD (GOLD score moderate (n.23), severe/very severe (n.33) Number of COPD medications, median: 5 Number of other medications, median: 5 **Comorbidities:** high blood pressure, osteoporosis, high cholesterol, diabetes, heart disease, depression: n.47 **Smoking:** 13 (current smoker), n. 43 (former smoker) **Ongoing pharmacological therapy**: N/D	**Type of intervention in telenursing:** standard care without the use of a tablet or the ability to monitor symptoms and physiological variables daily **Duration:** 12 months **Follow-up**: 3,6 and 12 months	**Adherence**: rated through MARS: from 22.4 to +0.33 (at 12 months) **Satisfaction**: N/D **Acceptability:** N/D
Fox et al., 2022 (29) *npi Primary care Respiratory Medicine* Prospective observational study United Kingdom No Funded	**Sample size**: 17 **Mean age/age range:** 62 **Gender ratio (M/F):** 7M/10F **Pulmonary disease**: diagnosis of asthma Exacerbation in the past 6 months: n.23 (n.16 patients) Hospital admissions: n.0 Out of hours attendance: n.3 ED attendance: n.3 **Comorbidities:** ≥ 1 (n.10), cardiovascular (n.7), gastrointestinal (n.6), diabetes (n.6) **Smoking:** n.5 (current smoker), n.9 (former smoker), n.3 (never smoker) **Ongoing pharmacological therapy**: MD	**Type of intervention in telenursing:** Specialized nurses evaluated the alerts generated by patient responses and provided telephone interventions (advice on inhaler use, therapy, specialist follow-up, etc.). **Digital tool used:** telephone **Duration:** 3 months **Follow-up**: 6 months **Other type of intervention**: N/D **Duration**: N/D **Follow-up**: N/D	**Adherence**: assessed using the Adherence starts with Knowledge: from 21.5 to 24.5 (n. 2 patients) **Satisfaction**: N/D **Acceptability:** N/D
	**Sample size**: 17 **Mean age/age range:** 65 **Gender ratio (M/F):** 8M/9F **Pulmonary disease**: diagnosis of COPD Exacerbations: n.37 Hospital admissions: n.3 Out of hours attendance: n.3 ED attendance: n.3 **Comorbidities:** ≥ 1 (n.14), cardiovascular (n.5), gastrointestinal (n.5), diabetes (n.4) **Smoking:** n.10 (current smoker), n.7 (former smoker), n.0 (never smoker) **Ongoing pharmacological therapy**: N/D	**Type of intervention in telenursing:** Specialized nurses evaluated the alerts generated by patient responses and provided telephone interventions (advice on inhaler use, therapy, specialist follow-up, etc.). **Digital tool used:** telephone **Duration:** 3 months **Follow-up**: 6 months **Other type of intervention**: N/D **Duration**: N/D **Follow-up**: N/D	**Adherence**: N/D **Satisfaction**: N/D **Acceptability:** N/D
	**Sample size**: 12 **Mean age/age range:** 64 **Gender ratio (M/F):** 1M/11F **Pulmonary disease**: diagnosis of asthma Exacerbation in the past 6 months: n.6 (n.12 patients) Hospital admissions: n.0 Out of hours attendance: n.0 ED attendance: n.0 **Comorbidities:** ≥ 1 (n.7), cardiovascular (n.5), gastrointestinal (n.2), diabetes (n.1) **Smoking:** n.5 (current smoker), n.6 (former smoker), n.1 (never smoker) **Ongoing pharmacological therapy**: N/D	**Type of intervention:** Standard care **Duration:** 3 months **Follow-up**: 6 months	**Adherence**: assessed using the Adherence starts with Knowledge: from 17 to 17.5 (n. 4 patients) **Satisfaction**: N/D **Acceptability:** N/D
	**Sample size**: 16 **Mean age/age range:** 69.5 **Gender ratio (M/F):** 8M/8F **Pulmonary disease**: diagnosis of COPD Exacerbation in the past 6 months: n.21 Hospital admissions: n.1 Out of hours attendance: n.4 ED attendance: n.0 **Comorbidities:** ≥ 1 (n.15), cardiovascular (n.9), gastrointestinal (n.6), diabetes (n.5) **Smoking:** n.6 (current smoker), n.10 (former smoker), n.0 (never smoker) **Ongoing pharmacological therapy**: N/D	**Type of intervention:** Standard care **Duration:** 3 months **Follow-up**: 6 months	**Adherence**: assessed using the Adherence starts with Knowledge: from 18.0 to 17.0 (n. 5 patients) **Satisfaction**: N/D **Acceptability:** N/D
Jakobsen et al., 2015 (30) *Telemed JE Health* RCT Danmark No funded	**Sample size**: 29 **Mean age/age range:** < 60(n.5), 60–70 (n.8), 70–80 (n.10), > 80 (n.6) **Gender Ratio (M/F**): 11M/18F **Pulmonary disease**: COPD (N/D severe or very severe according to GOLD score) Number of COPD re-admissions (6 months prior to trial): n.0 (n.17), n.1(n.6), > 1(n.6) **Comorbidities:** MD **Smoking:** n.16 (current smoker), n.12 (former smoker), n.1(never smoker) **Ongoing pharmacological therapy**: LTOT: n.1	**Type of intervention in telenursing:** Home hospitalization **Digital tool used:** touchscreen with a webcam and telemonitoring devices (pulse oximeter, spirometer, thermometer, nebulizer for aerosolized inhalation medication, oxygen compressor) **Duration:** 30 days **Follow-up**: 30, 60 and 180 days after discharge **Other type of intervention**: N/D **Duration**: N/D **Follow-up**: N/D	**Adherence**: N/D **Satisfaction**: assessed through non non-validated questionnaire (consisted of 29 questions: 24 questions were presented in a 5-point Likert- scale and 5 questions were close-ended (yes/no): completed by 20 patients **Acceptability:** N/D
	**Sample size**: 28 **Mean age/age range:** < 60 (n.5), 60–70 (n.8), 70–80 (n.9), > 80 (n.6) **Gender Ratio (M/F**): 11M/17F **Pulmonary disease**: COPD diagnosis (severe/very severe according to GOLD score) Number of COPD re-admissions 6 months prior to trial: n.0 (n.16), n.1(n.8), > 1(n.4) **Comorbidities:** N/D **Smoking:** n.14 (current smoker), n.14 (former smoker), n.0 (never smoker) **Ongoing pharmacological therapy**: LTOT: n.2	**Type of intervention:** Standard hospital treatment for an exacerbation **Duration:** 30 days **Follow-up**: 30, 60 and 180 days after discharge	**Adherence**: N/D **Satisfaction**: N/D **Acceptability:** N/D
Kamei et al., 2019 (31) *Nursing & Health Sciences* Prospective observational study Japan *Japanese Grants-in-Aid for Scientific Research* funded	**Sample size**: 24 **Mean age/age range:** 76.1 **Gender ratio (M/F):** 23M/1F **Pulmonary disease**: COPD (stage IV GOLD score) Exacerbation (in the past 12 weeks): n.10 **Comorbidities:** N/D n. 17 **Smoking:** MD **Ongoing pharmacological therapy**: MD	**Type of intervention in telenursing:** telemonitoring daily **Digital tool used:** touch panel and tablet PC **Duration:** 12 weeks **Follow-up**: 12 weeks **Other type of intervention**: N/D **Duration**: N/D **Follow-up**: N/D	**Adherence**: rated as data-transmission adherence (88.9% data transmitted) **Satisfaction**: not specific to COPD patients **Acceptability:** not specific to COPD patients
Lewis et al., 2010 (32) *J of COPD* Pilot RCT United Kingdom No funded	**Sample size**: 20 **Mean age/age range:** 67 **Gender ratio (M/F):** 10M/10F **Pulmonary disease**: diagnosis of COPD (moderate to severe) COPD related admissions (in the previous 12 months), median: n.0 **Comorbidities:** N/D 92% known **Smoking:** n.1 (current smoker) **Ongoing pharmacological therapy**: MD	**Type of intervention in telenursing:** telemonitoring of vital signs and symptoms via a dedicated platform, with response to alarms, telephone contact, and coordination with doctors for therapy adaptation (first 6 months) **Digital tool used:** telemonitoring devices (thermometer and oximeter), telephone and a portable device for data collection and transmission (connected to telephone) **Duration:** 12 months **Follow-up**: 6 months **Other type of intervention**: standard care **Duration**: 6 months **Follow-up**: 6 months	**Adherence**: very high (median of 97% of expected data loaded) **Satisfaction**: none of the 17 patients who completed telemonitoring reported any difficulties; 15/17 rated it as “useful” or “very useful.” **Acceptability:** N/D
	**Sample size**: 20 **Mean age/age range:** 70 **Gender ratio (M/F):** 10M/10F **Pulmonary disease**: diagnosis of COPD (moderate to severe) COPD related admissions (in the previous 12 months), median: n.0 **Comorbidities:** N/D 88% known **Smoking:** n.1 (current smoker) **Ongoing pharmacological therapy**: N/D	**Type of intervention:** standard care **Duration:** 12 months **Follow-up**: 6 months	**Adherence**: N/D **Satisfaction**: N/D **Acceptability:** N/D
Lewis et al**.**, 2010 (33) *J Telemed and Telecare* RCT United Kingdom No Funded	**Sample size**: 20 **Mean age/age range:** 70 **Gender Ratio (M/F**): 10M/10F **Pulmonary disease**: COPD diagnosis (moderate to severe) **Comorbidities:** N/D 92% **Smoking:** n.1 (current smoker) **Ongoing pharmacological therapy**: N/D	**Type of intervention in telenursing:** Home telemonitoring with twice-daily symptom reporting. Nurses can access the data via a website and receive email alerts if certain conditions are detected. **Digital tool used:** handheld telemonitor **Duration:** 12 months (6 months of telemonitoring and 6 months of standard care) **Follow-up**: daily **Other type of intervention**: N/D **Duration**: N/D **Follow-up**: N/D	**Adherence**: N/D **Satisfaction**: N/D **Acceptability:** N/D
	**Sample size**: 20 **Mean age/age range:** 73 **Gender Ratio (M/F**): 10M/10F **Pulmonary disease**: COPD diagnosis (moderate to severe) **Comorbidities:** N/D 88% **Smoking:** n.1 (current smoker) **Ongoing pharmacological therapy**: N/D	**Type of intervention:** Standard care **Duration:** 12 months	**Adherence**: N/D **Satisfaction**: N/D **Acceptability:** N/D
Mammen et al., 2020 (35) *J of Asthma* USA Prospective observational study No funded	**Sample size**: 30 **Mean age/age range**: 32.97 **Gender ratio (M/F):** 11 M/ 19F **Pulmonary disease**: Asthma according Expert Panel Report-3 (intermittent (n.2), mild (n.1), moderate (n.11), severe (n.16) Patient Asthma Control (well controlled (n.2), not well controlled (n.12), very poorly controlled (n.16) Preventive office visits for asthma, mean: 0.62 Asthma-related emergency visit (chart review): 0.70 **Smoking:** n.11(current smoker) **Comorbidities:** mental illness (n.17), substance use disorder (n.4) **Ongoing pharmacological therapy**: Prescriptions for controller medication (Inhaled corticosteroids, leukotriene receptor agonists), mean: 3.39; Prescriptions for short-acting beta agonist, mean: 4.0; Systemic corticosteroid use: 0.54	**Type of intervention in telenursing:** remote symptom monitoring, periodic nurse tele visits (every 2–6 weeks), self-management training through integrated educational modules, clinical decision support (CDS) software **Digital tool used:** Smartphone and a peak flow meter digital for Peak Expiratory Flow/ FEV_1_ **Duration:** 6 months **Follow-up**: 2–6 weeks and 2–3 months **Other type of intervention**: N/D **Duration**: N/D **Follow-up**: N/D	**Adherence:** increased from 45.58% to 85.29% **Satisfaction**: N/D **Acceptability**: 95.7% (assessed via survey and end-of-study interviews)
Mammen et al., 2019 (34) *J Telemed and Telecare* USA Prospective observational study Funded by *Sigma Theta Tau, Epsilon Xi*	**Sample size**: 7 **Mean age/age range:** 29.5 **Gender ratio (M/F):** 1M/6F **Pulmonary disease**: diagnosis of current Asthma n.0 (well-controlled), n.2 (not-well-controlled), n.5 (very poorly controlled) Emergency visits (year prior), mean: 0.28 **Comorbidities:** n.5 (mental illness), n.4 (substance use disorder) **Smoking:** N/D **Ongoing pharmacological therapy**: Short acting beta agonist (indicated as prescriptions from the previous year), mean: 4.29 Controller medication (indicated as prescriptions from the previous year), mean: 1.57 out of hours (indicated as prescriptions from the previous year), mean: 0.43	**Type of intervention in telenursing: r**emote symptom monitoring, telemedicine visits via smartphone, self-management training, clinical decision support **Digital tool used:** Smartphone, Zoom video consultation and digital peak flow meter to measure Peak Expiratory Flow/FEV_1_ **Duration:** 3 months **Follow-up**: Telemedicine visits were performed every 2 weeks until asthma control was achieved, then once a month. Participants had an average of 4 telemedicine visits in total. **Other type of intervention**: N/D **Duration**: N/D **Follow-up**: N/D	**Adherence**: Symptoms are recorded on average 32 days out of 90. Telemedicine visits: 68% completed as planned, with no-show and referral rates like outpatient visits. **Satisfaction**: average score 6.57/7 through the USE-Q and 1:1 interview (93.9% satisfaction) **Acceptability:** high (93.9%), assessed through 1:1 interview and the USE-Q (6/7 patients said the program was “life-changing”)
Mirón Rubio et al., 2018 (36) *Expert Review of Respiratory Medicine* Prospective observational study Spain *University, Hospital of Torrejón* funded	**Sample size**: 28 **Mean age/age range:** 78 **Gender ratio (M/F):** 26M/2F **Pulmonary disease**: COPD according to GOLD I (n.1), II (n.14), III (n.10), IV (n.3) COPD exacerbation (in the last year): N/D two or more **Comorbidities:** N/D **Smoking:** n.2 (current smoker) **Ongoing pharmacological therapy**: LTOT, n: 11	**Type of intervention in telenursing:** Telemonitoring nurse (programmed calls, programmed visits to the patient’s home and therapeutic recommendation) and home hospitalization. Nurses received a message in case of altered parameters and decided what to do **Digital tool used:** Telemonitoring devices (pulse-oximeter, heart rate monitor, blood pressure) and phone **Duration:** 6 months **Follow-up**: daily **Other type of intervention**: N/D **Duration**: N/D **Follow-up**: N/D	**Adherence**: N/D **Satisfaction**: N/D satisfaction questionnaire rate was high (mean 77%) **Acceptability:** N/D
	**Sample size**: 28 **Mean age/age range:** 78 **Gender ratio (M/F):** 26M/2F **Pulmonary disease**: COPD according to GOLD I (n.1), II (n.14), III (n.10), IV (n.3) COPD exacerbation (in the last year): N/D two or more **Comorbidities:** N/D **Smoking:** n.2 (current smoker) **Ongoing pharmacological therapy**: LTOT, n: 11	**Type of intervention:** usual care **Duration:** the same month of the previous year	**Adherence**: N/D **Satisfaction**: N/D **Acceptability:** N/D
Nemanic et al., 2018 (37) *J of Asthma* Slovenia RCT No funded	**Sample size**: 51 **Mean age/age range:** 45 **Gender ratio (M/F):** 24M/27F **Pulmonary disease**: Asthma diagnosis Acute exacerbation (in the last year): n.48 **Comorbidities:** MD **Smoking:** n.13(former smoker), n.7(current smoker), n.31 (non-smoker) **Ongoing pharmacological therapy**: Inhaled corticosteroids: n.13; Inhaled corticosteroids+long action beta-adrenergic agonist: n.37	**Type of intervention in telenursing:** home telemonitoring with information technology support and nursing management **Digital tool used:** mobile phone **Duration:** 12 months **Follow-up**: N/D **Other type of intervention**: N/D **Duration**: N/D **Follow-up**: N/D	**Adherence**: it was already high at baseline **Satisfaction**: N/D **Acceptability:** N/D
	**Sample size**: 49 **Mean age/age range:** 53 **Gender ratio (M/F):** 24M/25F **Pulmonary disease**: Asthma diagnosis Acute exacerbation (in the last year): n.35 **Comorbidities:** N/D **Smoking:** n.18 (former smoker), n.6 (current smoker), n.25 (non-smoker) **Ongoing pharmacological therapy**: Inhaled corticosteroids: n.13; Inhaled corticosteroids+long action beta-adrenergic agonist: n.35	**Type of intervention:** standard care **Duration:** 12 months **Follow-up**: 12 months	**Adherence**: N/D **Satisfaction**: N/D **Acceptability:** N/D
Pak-Chun Chau et al., 2012 (24) *Int J Med Inform* RCT Hong Kong No funded	**Sample size**: 22 **Mean age/age range:** 73.50 **Gender Ratio (M/F**): 21M/1F **Pulmonary disease**: COPD diagnosis: moderate (n.4), severe (n.9), very severe (n.9) Number of re-admissions for COPD exacerbation over the past year: 2.41 Average length of hospital stays due to COPD exacerbation over the past year: 7.66 **Comorbidities:** N/D **Smoking:** N/D **Ongoing pharmacological therapy**: N/D	**Type of intervention in telenursing:** daily monitored the data that was transmitted by the patients and called them to discuss the results, provide advice/support **Digital tool used: a** small portable unit, telemonitoring devices (blood pressure, weight, temperature, pulse, oxygen saturation level) and phone **Duration:** 2 months **Follow-up**: 2 months **Other type of intervention**: N/D **Duration**: N/D **Follow-up**: N/D	**Adherence**: 79% transmitted their oxygen saturation data 3 times a day, 60% transmitted their respiratory rates, about 98% of the participants transmitted their oxygen saturation data once a day, 83% transmitted their respiratory rates **Satisfaction**: detected by the User Satisfaction Questionnaire (91% of participants were satisfied with the telecare service and 100% were satisfied with the support from the nurse offering the service) **Acceptability:** N/D
	**Sample size**: 18 **Mean age/age range:** 72.22 **Gender Ratio (M/F**): 18M/0F **Pulmonary disease**: COPD diagnosis: moderate (n.6), severe (n.4), very severe (n.8) Number of re-admissions for COPD exacerbation over the past year: 2.89 Average length of hospital stays due to COPD exacerbation over the past year: 8.06 **Comorbidities:** N/D **Smoking:** N/D **Ongoing pharmacological therapy**: N/D	**Type of intervention:** education program on self-care and symptom management techniques **Duration:** 2 months **Follow-up**: 2 months	**Adherence**: N/D **Satisfaction**: N/D **Acceptability:** N/D
Paré et al., 2013 (38) *Elsevier Masson* RCT Canada No Funded	**Sample size**: 60 **Mean age/age range:** 67.8 **Gender ratio (M/F):** 19M/41F **Pulmonary disease**: diagnosis of severe COPD. ER visits (in the past 365 days): n.121 ER visits (at least one in the past 365 days): n.44 patients Hospital admissions (in the past 365 days): n.88 Length of stay (in the past 365 days): n.908 days Home visits by nurses (in the past 365 days): n. 559 **Comorbidities:** n.15 has at least one related diagnosis (heart failure, diabetes, hypertension, fibrosis, etc.) **Smoking:** N/D **Ongoing pharmacological therapy**: HOT: n.37	**Type of intervention in telenursing:** Nurse case managers monitored patient-reported data daily, responded to automatic alarms, educated patients, and intervened promptly to correct deviations from therapy or clinical deteriorations. **Digital tool used:** touchscreen with an integrated modem capable of monitoring various parameters and phone **Duration:** 21.5 months (12 months pre-intervention, 6 months during, 3.5 months post-intervention). **Follow-up**: 6 and 3.5 months after intervention **Other type of intervention**: N/D **Duration**: N/D **Follow-up**: N/D	**Adherence**: N/D **Satisfaction**: evaluated with a series of questions whose results indicate very high satisfaction among most respondents, with an overall average of 4.0 on a scale of 5 (where 1 = not at all and 5 = enormously) **Acceptability:** N/D
	**Sample size**: 60 **Mean age/age range:** 68.6 **Gender ratio (M/F):** 19M/41F **Pulmonary disease**: diagnosis of severe COPD ER visits (in the past 365 days): n.127 ER visits (at least one in the past 365 days): n.49 patients Hospital admissions (in the past 365 days): n.104 LOS (in the past 365 days): n.843 days Home visits by nurses (in the past 365 days): n. 583 **Comorbidities:** n.16 has at least one related diagnosis (heart failure, diabetes, hypertension, fibrosis, etc.) **Smoking:** N/D **Ongoing pharmacological therapy**: HOT: n.36	**Type of intervention:** Traditional home care (without telemonitoring) **Duration:** 21.5 months (12 months pre-intervention, 6 months during, 3.5 months post-intervention). **Follow-up**: 6 and 3.5 months after intervention	**Adherence**: N/D **Satisfaction**: N/D **Acceptability:** N/D
Persson et al., 2019 (50) *International J COPD* Prospective observational study Sweden *The country council of Östergötland, the European Regional Development Fund and RISE Research Institutes of Sweden* funded	**Sample size**: 36 **Mean age/age range:** 75 ± 6/65–86 **Gender ratio (M/F):** 14M/22F **Pulmonary disease**: diagnosis of COPD according GOLD stage: n.3 (I-II), n.7 (III), n.26 (IV) Time hospitalized, all causes (12 months prior to inclusion): 3.4 ± 1.8 (per subject) **Comorbidities:** scored using Charlson Comorbidity Index: 0–1 (n.5), 2 (n.9, 3 (n.12), ≥ 4 (n.10) **Smoking:** n.10 (current smoker), n.25 (former smoker), n.0 (never smoker) **Ongoing pharmacological therapy**: LTOT: n. 7	**Type of intervention in telenursing:** Hospital-based home care based on data acquired through a telemonitoring system. Based on the acquired data, healthcare workers were responsible for both monitoring the subjects and taking the medical actions they took. **Digital tool used:** digital pen, phone and health diary module where patients entered vital signs (bool pressure, hate rate, saturation and temperature) measured with different devices **Duration:** 12 months **Follow-up**: daily **Other type of intervention**: hospital-based home care **Duration**: 12 months **Follow-up**: 12 months	**Adherence**: N/D **Satisfaction**: evaluated through non validated questionnaires at 1,6 and 12 months. The collective responses reflected a great satisfaction with the telemonitoring system used and showed a positive impact on the subjects’ own experience of their health status. **Acceptability:** N/D
Pinnock et al., 2013 (51) *BMJ* RCT United Kingdom Funded by an *NHS applied research programme grant from the Chief Scientist Office of the Scottish Government*	**Sample size**: 128 **Mean age/age range:** 69.4 **Gender ratio (M/F):** 53M/75F **Pulmonary disease**: diagnosis of COPD according GOLD stage: n.46 (mild-moderate), n.45 (severe), n.37 (very severe) **Comorbidities:** N/D one or more comorbid conditions n.78 **Smoking:** n.37 (current smoker), n. 89 (former smoker), n.2 (never smoker) **Ongoing pharmacological therapy**: N/D	**Type of intervention in telenursing:** telemonitoring daily. In case of alarm rates or significantly worsened symptoms, the nurse received an alert email and contacted the patient by telephone and communicated with doctors for shared management of therapies. **Digital tool used:** touch screen, telemonitoring devices (oximeter and thermometer), and telephone **Duration:** 12 months **Follow-up**: 12 months **Other type of intervention**: education program on self-management of exacerbation, written management plans, and emergency supply of antibiotics and steroids **Duration**: 12 months **Follow-up**: N/D	**Adherence**: rated through MARS: from 24.0 (at baseline) to 24.0 (at 12 months) on a sample of 104 patients. **Satisfaction**: N/D **Acceptability:** N/D
	**Sample size**: 128 **Mean age/age range:** 69.4 **Gender ratio (M/F):** 63M/65F **Pulmonary disease**: COPD according GOLD stage: n.42 (mild-moderate), n.42 (severe) n.44 (very severe) **Comorbidities:** N/D one or more comorbid conditions n.91 **Smoking:** n.30 (current smoker), n. 98 (former smoker), n.0 (never smoker) **Ongoing pharmacological therapy**: N/D	**Type of intervention:** standard care and a program of education on self-management of exacerbation, reinforced by a written management plan and an emergency supply of antibiotics and steroids **Duration:** 12 months **Follow-up**: 12 months	**Adherence**: rated through MARS: from 23.6 (at baseline) to 23.7 (at 12 months) on a sample of 101 patients **Satisfaction**: N/D **Acceptability:** N/D
Prabhakaran et al., 2019 (39) *BMJ Health & Care Information* Singapore RCT *Singapore National Asthma Program* funded	**Sample size**: 212 **Mean age/age range:** 37.1 **Gender ratio (M/F):** 85M/127F **Pulmonary disease**: diagnosis of Asthma Asthma control, n: poor (5–12) (n.106), partial (15–19) (n.106) **Comorbidities, number:** 0 (n.181), 1(n.21), 2 (n.6), 3(n.2),4 (n.1),5 (n.1) **Smoking:** n.54 (current smoker) **Ongoing pharmacological therapy**: N/D	**Type of intervention in telenursing:** daily and weekly SMS reminders for monitoring post-discharge support, which included remote nursing management to evaluate drug therapy or the search for healthcare **Digital tool used:** short message service and email **Duration:** daily monitoring (1–2 weeks), then weekly (3 weeks); 2-year study duration **Follow-up**: 5 weeks and 3 months **Other type of intervention**: N/D **Duration**: N/D **Follow-up**: N/D	**Adherence**: N/D **Satisfaction**: rated at 5 weeks referred to short message service: strongly disagree (n.0), disagree (n.4), neutral (n.4), agree (n.137), strongly agree (n.21) **Acceptability:** N/D
	**Sample size**: 212 **Mean age/age range:** 40.5 **Gender ratio (M/F):** 95M/117F **Pulmonary disease**: diagnosis of asthma **Comorbidities, number:** 0 (n.170), 1 (n.26), 2 (n.13), 3 (n.1),4 (n.2),5 (n.0) **Smoking:** n.64 (current smoker) **Ongoing pharmacological therapy**: N/D	**Type of intervention:** standard care **Duration:** 2 years **Follow-up**: 5 weeks and 3 months	**Adherence**: N/D **Satisfaction**: N/D **Acceptability:** N/D
Roberts et al., 2008 (54) *Intern Med J* Australia Prospective observational study No Funding	**Sample size:** 118 **Mean age/age range:** 69 ± 8 **Gender ratio (M/F):** 72M/46F **Pulmonary disease:** COPD COPD hospitalizations (in the last year): 1.0 ± 1.5 **Comorbidities:** ischemic heart disease (n.34), diabetes mellitus (n.11) **Smoking:** N/D **Ongoing pharmacological therapy:** O_2_ therapy (%): 31	**Type of intervention in telenursing**: 24/7 nurse-led hotline, access to personalized action plans, electronic medical record-based triage, Self-management education and symptom assessment, coordination with emergency services and scheduled hospitalizations **Digital tool used:** mobile phone and laptop **Duration:** 26 months **Follow-up:** 26 months **Other type of intervention**: ambulatory care respiratory program **Duration**: continuous access **Follow-up**: based on patient need	**Adherence**: N/D **Satisfaction**: N/D **Acceptability:** N/D
Saleh et al., 2023 (40) *Multidisciplinary Respiratory Medicine* Norway RCT Funded by *the Western Norway Regional Heath Authority research fund* funded	**Sample size**: 57 **Mean age/age range**: 69.0 **Gender ratio (M/F):** 15M/42F **Pulmonary disease**: COPD (GOLD stage III-IV) Number of admissions in the past year: 26 **Smoking:** n.13(current smoker), n.57 (former smoker) **Comorbidities:** cardiovascular disease (n.20), heart failure (n.3), depression (n.17), osteoporosis (n.9) **Ongoing pharmacological therapy**: LTOT at home: n.6; NIV at home: n.1	**Type of intervention in telenursing:** Daily home video nursing consultations for two weeks post-discharge, with oxygen saturation and heart rate monitoring, and educational and clinical consultations by respiratory nurse specialists **Digital tool used:** Smartphone **Duration:** 2 weeks **Follow-up**: 3 weeks post-discharge, at 6 and 12 months **Other type of intervention**: N/D **Duration**: N/D **Follow-up**: N/D	**Adherence:** N/D **Satisfaction**: assessed via a non-validated questionnaire (46/57 patients reported feeling safe; 44/57 found that video nursing consultations had great importance for the further management of their COPD -related problems; 55/57 found the telemedicine equipment easy to operate; all patients recommend telemedicine for follow up after a COPD exacerbation) **Acceptability**: N/D
	**Sample size**: 59 **Mean age/age range:** 68.64 **Gender ratio (M/F):** 21M/38F **Pulmonary disease**: COPD (GOLD stage III-IV) Number of admissions in the past year: 25 **Comorbidities:** cardiovascular disease (n.27), heart failure (n.10), depression (n.14), osteoporosis (n.16) **Smoking:** n.17 (current smoker), n.59 (former smoker) **Ongoing pharmacological therapy**: LTOT at home: n.15; NIV at home: n.5	**Type of intervention in telenursing:** Phone calls **Digital tool used:** Telephone **Duration:** 2 weeks **Follow-up**: 3 weeks post-discharge, at 6 and 12 months **Other type of intervention**: N/D **Duration**: N/D **Follow-up**: N/D	**Adherence**: N/D **Satisfaction**: assessed via a non-validated questionnaire (26/59 found that phone calls had great importance for the further management of their COPD-related problems) **Acceptability:** N/D
	**Sample size**: n.57 **Mean age/age range:** 68.07 **Gender ratio (M/F):** 26M/31F **Pulmonary disease**: COPD (GOLD stage III-IV) Number of admissions in the past year: 20 **Comorbidities:** cardiovascular disease (n.25), heart failure (n.6), depression (n.16), osteoporosis (n.14) **Smoking:** n.55 (former smoker), n.17(current smoker) **Ongoing pharmacological therapy**: LTOT at home: n.10; NIV at home: n.3	**Type of intervention:** Best Standard Practice COPD care **Duration:** 2 weeks **Follow-up**: 3 weeks post-discharge, 6 and 12 months	**Adherence**: N/D **Satisfaction**: N/D **Acceptability:** N/D
Saleh et al., 2014 (41) *Multidisciplinary Respiratory Medicine* Retrospective observational study Norway No funded	**Sample size**: 99 **Mean age/age range:** 60.6 **Gender Ratio (M/F**): 45M/54F **Pulmonary disease**: COPD diagnosis (n.27 GOLD IV) **Comorbidities:** cardiovascular disease (n.51), depression (n.40) **Smoking:** n.27 (current smoker), n.69 (former smoker) **Ongoing pharmacological therapy**: NIV at home: n.15; LTOT at home: n.15; Bronchodilators (nebulized) (ipratropium and salbutamol): n.99/long-acting beta-2-agonist; Corticosteroids: n.96; Antibiotics: n.94	**Type of intervention in telenursing:** the nurse performed clinical observations according to a checklist, measured oxygen saturation, and advised the patient on how to manage COPD-related symptoms, medication use, and how to maintain normal daily activities and physical activity. **Digital tool used:** computer equipped with a webcam and microphone, and telemonitoring devices (oxygen saturation, heart rate, spirometry). The computer had two buttons: one to contact the nurse based on daily TVC appointments on weekdays during the day, and an alarm button to press in case of urgent consultation, available 24/7. **Duration**: 2 weeks **Follow-up**: 6 and 12 months after TVC **Other type of intervention**: N/D **Duration**: N/D **Follow-up**: N/D	**Adherence**: N/D **Satisfaction**: detected through a survey (response rate n.89–90%). n.47 (52.4%) they generally felt safe or very safe when discharged from the hospital without TVC; n.80 (90.5%) safety when discharged to TVC at home, *p* < 0.001; n.43 (48%) found that TVC had great importance for their further management of their COPD related problems; n.85 (96%) found the telemedicine equipment easy to operate; n.85 (95.2%) reported that they handled the tele-video conferencing system on their own. N.70 (78.7%) preferred TVC over only phone calls **Acceptability:** N/D
Scalvini et al., 2016 (42) *J of Telemedicine and Telecare* Prospective observation study Italy No funding	**Sample size**: 530 **Mean age/age range:** 74 ± 6 **Gender ratio (M/F):** 387/143 **Pulmonary disease**: COPD (GOLD class III-IV) and chronic respiratory insufficiency Number of admissions to hospital (in the previous 12 months): N/D (n.530 one or two severe relapses for acute COPD) **Comorbidities:** ND one or more **Smoking:** N/D **Ongoing pharmacological therapy**: NIV: n.530; LTOT at home: n.530 **Quality of life parameters:** MD	**Type of intervention in telenursing:** nurse-managed telephone support and telemonitoring service provided to the patient in their home **Digital tool used:** pulse oximeter device and telephone **Duration:** 6 to 12 months **Follow-up**: N/D **Other type of intervention**: N/D **Duration**: N/D **Follow-up**: N/D	**Adherence**: N/D **Satisfaction**: Very high (n.424–80%) **Acceptability:** N/D
Segrelles Calvo et al., 2014 (55) *Respir Med* Spain RCT *Linde Healthcare* funded	**Sample size**: 29 **Mean age/age range**: 75 ± 9.7 **Gender ratio (M/F):** 22M/7F **Pulmonary disease**: COPD according GOLD score (n.29) Dyspnea II (n.3); III (n.17); IV (n.9) COPD hospitalizations (in the last year): n.30 times **Smoking:** former smokers for at least 6 months (n.29) **Comorbidities:** N/D multiple diseases **Ongoing pharmacological therapy**: O_2_ therapy, hours/day: 18.6 ± 3.8 Long action muscarinic antagonist+long action beta-adrenergic agonist+inhaled cortico-steroids: 26; phosphodiesterase 4 inhibitor: 2; Mucolythics: 11; Theophyllines: 2; Oral steroides: 1	**Type of intervention in telenursing:** Telemonitoring daily **Digital tool used:** Telemonitoring devices (spirometer, pulse-oximeter, heart rate monitor) + telephone calls **Duration:** 7 months **Follow-up**: daily **Other type of intervention**: N/D **Duration**: N/D **Follow-up**: N/D	**Adherence:** average of telemonitoring days was 152.2 days/7months (72.5%) **Satisfaction**: N/D satisfaction questionnaire rate through telephone calls was high (mean 72.5%) **Acceptability**: MD
	**Sample size**: 30 **Mean age/age range:** 72,7 ± 9,3 **Gender ratio (M/F):** 22M/8F **Pulmonary disease**: COPD according GOLD score (n.30) Dyspnea II (n.8); III (n.3); IV (n.17) COPD hospitalizations (in the last year): n.29 times **Comorbidities:** N/D multiple diseases **Smoking:** former smokers for at least 6 months (n.30) **Ongoing pharmacological therapy**: O_2_ therapy, hours/day: 20.2 ± 4.7; Long action muscarinic antagonist+long action beta-adrenergic agonist+inhaled cortico-steroids: 23; phosphodiesterase 4 inhibitor: 6; Mucolytics: 12; Theophyllines: 3; Oral steroids: 4	**Type of intervention:** Standard follow-up **Duration:** 7 months **Follow-up**: 7 months	**Adherence**: N/D **Satisfaction**: N/D **Acceptability:** N/D
Shany et al., 2016 (43) *J Telemed Telecare* RCT Australia Funded by *the Department of State and Regional Development of New South Wales Government, TeleMedCare, Australian Research Council, Sydney West Area Health Service and University of New South Wales*	**Sample size**: 21 **Mean age/age range:** 72.1 ± 7.5 **Gender ratio (M/F):** 10M/11F **Pulmonary disease**: COPD diagnosis. Number of admissions to hospital (prior to study): n. 64 Sum of length of stay for all admissions (days) (prior to study): n.648 Length of stay per admission (days) (prior to study): n.9 Number of ED presentations (prior to study): n.82 **Comorbidities:** not specified **Smoking:** N/D **Ongoing pharmacological therapy**: N/D	**Type of intervention in telenursing:** Home telemonitoring **Digital tool used:** Telemonitoring devices (oximetry, temperature, pulse, electrocardiogram, blood pressure, spirometry and weight) and telephone **Duration:** 12 months **Follow-up**: 12 months **Other type of intervention**: home visits **Duration**: 12 months **Follow-up**: 12 months	**Adherence**: N/D **Satisfaction**: N/D **Acceptability:** N/D
Shimoyama et al., 2023 (44) *PLoS ONE* Japan RCT No funded	**Sample size**: 15 **Mean age/age range**: 72.5 ± 10.9 **Gender ratio (M/F):** 9M/6F **Pulmonary disease**: COPD (n.6), Pulmonary cell hypoventilation syndrome (n.5), Pulmonary tuberculosis sequelae (n. 2) Number of hospitalizations in the past 3 months (times), median: 0.0 Number of days hospitalized in the past 3 months (days), median: 0.0 Number of unscheduled outpatient visits in the past 3 months (times), median: 0.0 **Smoking:** n.10(former smoker) n.0(current smoker) **Comorbidities:** scoliosis (n.1), spinal caries (n.1) **Ongoing pharmacological therapy**: duration of oxygen therapy, mean: non-invasive positive pressure ventilation (years):2.9, HOT (years):1.7; HOT, *n* (%): 8; non-invasive positive pressure ventilation treatment schedule, n: 15 (nocturnal)	**Type of intervention in telenursing:** Telemonitoring daily, telehealth counselling and personalised health education **Digital tool used:** Videophone (n.13), phone (n.12) and email (n.10) **Duration:** 3 months **Follow-up**: daily **Other type of intervention**: N/D **Duration**: N/D **Follow-up**: N/D	**Adherence:** N/D **Satisfaction**: N/D **Acceptability**: N/D
	**Sample size**: n.16 **Mean age/age range:** 73.6 ± 10.0 **Gender ratio (M/F):** 9M/7F **Pulmonary disease**: COPD (n.7), Pulmonary cell hypoventilation syndrome (n.5), Pulmonary tuberculosis sequelae (n. 4) Number of hospitalizations in the past 3 months (times), median: 0.0 Number of days hospitalized in the past 3 months (days), median: 0.0 Number of unscheduled outpatient visits in the past 3 months (times), median: 0.0 **Comorbidities:** N/D **Smoking:** n.9 (former smoker), n.1(current smoker) **Ongoing pharmacological therapy**: Duration of oxygen therapy, mean: non-invasive positive pressure ventilation (years): 3.2, HOT (years): 2.3; HOT, n: 8; non-invasive positive pressure ventilation treatment schedule, n: 16 (nocturnal)	**Type of intervention:** traditional care **Duration:** 3 months **Follow-up**: 3 months	**Adherence**: N/D **Satisfaction**: N/D **Acceptability:** N/D
Sorknæs et al**.**, 2011 (45) *The Clinical Respiratory Journal* Prospective observational study Denmark Funded by *the European Commission, ICT for Citizens and Businesses, eTEN, Better Breathing, Project Number 045225 and partly by the Region of Southern Denmark*	**Sample size**: 50 **Mean age/age range:** 74.5 **Gender Ratio (M/F**): 20M/30F **Pulmonary disease**: COPD diagnosis Number of days admitted with exacerbation in COPD (the last two years): 1 **Comorbidities:** N/D **Smoking:** n.11 (current smoker) **Ongoing pharmacological therapy**: LTOT: n.8	**Type of intervention in telenursing:** Post-discharge nursing teleconsultations (i.e., dyspnoea, general condition, physical activity and anxiety), measured saturation and lung function and informed the patient how to prevent exacerbations and how to use medications. **Digital tool used:** a computer with web camera, a microphone and measurement equipment (oxygen saturation and spirometry) **Duration:** 28 days **Follow-up**: daily **Other type of intervention**: N/D **Duration**: N/D **Follow-up**: N/D	**Adherence**: N/D **Satisfaction**: assessed trough a questionnaire with seven questions (high but not quantifiable) **Acceptability:** N/D
	**Sample size**: 50 **Mean age/age range:** 74.5 **Gender Ratio (M/F**): 23M/27F **Pulmonary disease**: COPD diagnosis Number of days admitted with exacerbation in COPD (in the last two years): 1.5 **Comorbidities:** N/D **Smoking:** n.23 (current smoker) **Ongoing pharmacological therapy**: LTOT: n.7	**Type of intervention:** standard care **Duration:** 28 days **Follow-up**: 28 days	**Adherence**: N/D **Satisfaction**: N/D **Acceptability:** N/D
Sorknaes et al., 2013 (52) *J Telemed and Telecare* RCT Denmark Partly funded by *the European Commission, the Danish Health Foundation, the Danish Nurses’ Organization, the University of Southern Denmark, the OUH-Odense University Hospital and Svendborg Hospital*	**Sample size**: 132 **Mean age/age range:** 71 **Gender ratio (M/F):** 53M/79F **Pulmonary disease**: diagnosis of COPD Hospital admissions (in the previous year), mean: 2.75 per patient (total), 2.27 per patient with acute exacerbation of COPD **Comorbidities:** infections (n.69), heart disease (n.46), cerebrovascular diseases (n.12), depression (n.2), diabetes (n.18), osteoporosis (n.22), cancer (n.0) **Smoking:** n.48(current smoker), n. 78 (former smoker), n.4 (never smoker) **Ongoing pharmacological therapy**: LTOT: n.11	**Type of intervention in telenursing:** Hospital nurses conducted daily video consultations, used a structured checklist for clinical assessments, educated patients on disease management and treatments, and coordinated any urgent medical interventions. **Digital tool used:** computer, webcam, microphone, telemonitoring devices (spirometer, pulse oximeter) and telephone **Duration:** 26 weeks **Follow-up**: 4, 8, 12 and 26 weeks **Other type of intervention**: N/D **Duration**: N/D **Follow-up**: N/D	**Adherence**: N/D **Satisfaction**: N/D **Acceptability:** N/D
	**Sample size**: 134 **Mean age/age range:** 72 **Gender ratio (M/F):** 51M/83F **Pulmonary disease**: diagnosis of COPD Hospital admissions (in the previous year), mean: 2.64 per patient (total), 2.20 per patient with acute exacerbation of COPD **Comorbidities:** infections (n.74), heart disease (n.48), cerebrovascular diseases (n.11), depression (n.3), diabetes (n.15), osteoporosis (n.26), cancer (n.1) **Smoking:** n.46(current smoker), n. 85 (former smoker), n.3(never smoker) **Ongoing pharmacological therapy**: LTOT: n.15	**Type of intervention:** standard care according to GOLD guidelines **Duration:** 5–9 days after discharge **Follow-up**: 4, 8, 12 and 26 weeks **Other type of intervention**: N/D **Duration**: N/D **Follow-up**: N/D	**Adherence**: N/D **Satisfaction**: N/D **Acceptability:** N/D
Te-Wei Ho et al., 2016 (49) *Nature* RCT Taiwan No funding	**Sample size**: 53 **Mean age/age range:** 81.4 ± 7.8 **Gender ratio (M/F):** 43M/10F **Pulmonary disease**: diagnosis of COPD according GOLD stage: n.35 (mild-moderate), n.18 (severe/very severe) Exacerbation admission (in the previous year): n.16 ER visit (in the previous year): n.19 **Comorbidities:** coronary artery disease (n.12), heart failure (n.14), hypertension (n.28), diabetes mellitus (n.11) **Smoking:** 58 ± 43 (pack-years) **Ongoing pharmacological therapy**: Short-acting *β*_2_ agonist: n.47; Long- acting β_2_ agonist: n.32; Long- acting anticholinergic: n.36; Inhaled corticosteroid: n.33	**Type of intervention in telenursing:** Home telemonitoring with nurses managing patient call systems and assessing whether emergency intervention was necessary **Digital tool used:** electronic diary, phone and telemonitoring devices (pulse oximeter, a thermometer and a sphygmomanometer) **Duration:** 6 months **Follow-up**: 2 months **Other type of intervention**: usual care from their primary care physicians **Duration**: N/D **Follow-up**: N/D	**Adherence**: N/D **Satisfaction**: N/D **Acceptability:** N/D
	**Sample size**: 53 **Mean age/age range:** 79.0 ± 9.6 **Gender ratio (M/F):** 38M/15F **Pulmonary disease**: diagnosis of COPD according GOLD stage: n.34 (mild-moderate), n.19 (severe/very severe) Exacerbation admission (in the previous year): n.19 ER visit (in the previous year): n.17 **Comorbidities:** coronary artery disease (n.9), heart failure (n.13), hypertension (n.33), diabetes mellitus (n.10) **Smoking:** 47 ± 31 (pack-years) **Ongoing pharmacological therapy**: Short-acting β_2_ agonist: n.45; Long-acting β_2_ agonist: n.35; Long-acting anticholinergic: n.34; inhaled corticosteroid: n.37	**Type of intervention:** usual care **Duration:** 6 months after discharge **Follow-up**: N/D	**Adherence**: N/D **Satisfaction**: N/D **Acceptability:** N/D
Trappenburg et al., 2008 (57) *Telemed J E Health* Netherlands Prospective observational study *Dutch Asthma Foundation (Astmafonds*) funded	**Sample size:** 59 **Mean age/age range:** 69 ± 8 **Gender ratio (M/F):** 27M/32F **Pulmonary disease:** COPD (GOLD stage III-IV) COPD hospitalizations (in the past 6 months): n.0 (n.32 patients), n.1 (n.17 patients), n.≥ 2 (n.10 patients) Exacerbations (in the past 6 months): n. 1.00 ± 1.45 ER visit (in the past 6 months): n. 0.13 ± 0.4 LOS (in the past 6 months): 9.1 ± 20.6 days Outpatients’ visits (in the past 6 months): n. 3.5 ± 2.0 **Comorbidities:** N/D **Smoking:** n.38 (former smoker) n.17(current smoker) **Ongoing pharmacological therapy:** Inhaled steroids: never (n.40), in episodes (n.10), always (n.9); Oral steroids: never (n.40), in episodes (n.14), always (n.5); Antibiotics: never (n.45), in episodes (n.13), always (n.1); Bronchodilators (short-acting): never (n.19), in episodes (n.9), always (n.31); Bronchodilators (long acting): never (n.16), in episodes (n.13), always (n.30)	**Type of intervention in telenursing**: home-based telemonitoring with structured daily questions (symptoms, medication use, education), nurse review and reactive telephone calls by a respiratory nurse (Monday to Friday) **Digital tool used:** a telephone and a large screen with 4 buttons for response about disease symptoms, medication compliance and knowledge and provide education about their condition. **Duration:** 6 months **Follow-up:** 6 months **Other type of intervention**: N/D **Duration**: N/D **Follow-up**: N/D	**Adherence**: N/D **Satisfaction**: N/D **Acceptability:** N/D
	**Sample size:** 56 **Mean age/age range:** 70 ± 10 **Gender ratio (M/F):** 34M/22F **Pulmonary disease:** COPD (GOLD stage III-IV) COPD hospitalizations (in the past 6 months): n.0 (72%), n.1 (12%), n.2 (4%) Exacerbations (in the past 6 months): n. 0.69 ± 1.32 ER visit (in the past 6 months): n. 0.02 ± 0.1 LOS (in the past 6 months): 6.56 ± 14.3 days Outpatients’ visits (in the past 6 months): n. 2.1 ± 1.7 **Comorbidities:** N/D **Smoking:** n.36 (former smoker) n.12(current smoker) **Ongoing pharmacological therapy:** inhaled steroids: never (n.38), in episodes (n.5), always (n.13); Oral steroids: never (n.42), in episodes (n.10), always (n.4); Antibiotics: never (n.48), in episodes (n.6), always (n.2); bronchodilators (short-acting): never (n.18), in episodes (n.5), always (n.33); bronchodilators (long acting): never (n.24), in episodes (n.10), always (n.22)	**Type of intervention**: outpatient COPD management without telemonitoring **Duration:** 6 months **Follow-up:** 6 months	**Adherence**: N/D **Satisfaction**: N/D **Acceptability:** N/D
Van der Meer et al., 2009 (46) *Ann intern med* RCT Netherlands Funded by *Netherlands Organization for Health Research and Development, ZonMw, and Netherlands Asthma Foundation*	**Sample size**: 101 **Mean age/age range:** 36/19–50 **Gender ratio (M/F):** 32M/69F **Pulmonary disease**: diagnosis of asthma according to International Classification of Primary Care Symptom-free days, %: 44.9 **Comorbidities:** MD **Smoking:** n.12 (current smoker), n. 30 (former smoker), n.58 (never smoker) **Ongoing pharmacological therapy**: Inhaled corticosteroid dose, mean: 497 (daily); Inhaled long-acting ß_2_- agonist use: 59%	**Type of intervention in telenursing:** Asthma nurses provided online communication, weekly feedback on asthma control scores, supervised automatic medication changes, responded to patient alerts, and delivered personalized education through group sessions and interactive content. **Digital tool used:** computer with a website and mobile phone **Duration:** 12 months **Follow-up**: 3 and 12 months **Other type of intervention**: N/D **Duration**: N/D **Follow-up**: N/D	**Adherence**: detected as self-reported medication adherence: from 6.46 (n.99 patients) to 6.32 (n.91 patients) **Satisfaction**: N/D **Acceptability:** N/D
	**Sample size**: 99 **Mean age/age range:** 37/18–50 **Gender ratio (M/F):** 29M/70F **Pulmonary disease**: diagnosis of asthma according to the International Classification of Primary Care Symptom-free days, %: 44.5 **Comorbidities:** N/D **Smoking:** n.14 (current smoker), n. 33 (former smoker), n.53 (never smoker) **Ongoing pharmacological therapy:** inhaled corticosteroid dose, mean: 517 (daily); inhaled long-acting ß_2_- agonist use: 60%	**Type of intervention in telenursing:** usual physician-provided care alone according to the Dutch general practice guidelines on asthma management in adults **Duration:** 12 months **Follow-up**: 3 and 12 months	**Adherence**: detected as self-reported medication adherence: from 6.19 (n.91 patients) to 6.37 (n.92 patients) **Satisfaction**: N/D **Acceptability:** N/D
Vitacca et al**.**, 2010 (47) *Eur Respir J* RCT Italy No Funding	**Sample size**: 118 **Mean age/age range:** 61.2 ± 17.6 **Gender Ratio (M/F**): 75M/43F **Pulmonary disease**: chronic respiratory failure: n. 57 (COPD), n.14(restrictive), n.24 (neuromuscular disease), n.12 (amyotrophic lateral sclerosis), n.11 (other) **Comorbidities:** 1.69 ± 1.4 N/D **Smoking:** n.7 (current smoker), n.55 (former smoker) **Ongoing pharmacological therapy**: invasive mechanic ventilation: n.8 (COPD), n.18 (other diagnosis); LTOT: n.75; NIV: n.21 (COPD), n.29 (other diagnosis)	**Type of intervention in telenursing:** real-time tele-consultation and the compilation of a clinical scoring system to assess any clinical variation **Digital tool used:** pulse oximeter device and telephone **Duration:** 12 months **Follow-up**: 12 months **Other type of intervention**: N/D **Duration**: N/D **Follow-up**: N/D	**Adherence**: N/D **Satisfaction**: N/D **Acceptability:** N/D
	**Sample size**: 102 **Mean age/age range:** 61.1 ± 17.4 **Gender Ratio (M/F**): 74M/28F **Pulmonary disease**: chronic respiratory failure: n. 44 (COPD), n.14(restrictive), n.26 (neuromuscular disease), n.10 (amyotrophic lateral sclerosis), n.8(other) **Comorbidities:** 1.57 ± 1.24 N/D **Smoking:** n.9 (current smoker), n.43 (former smoker) **Ongoing pharmacological therapy**: NIV: n.52; invasive mechanical ventilation: n.21; LTOT: n.63	**Type of intervention:** standard care with follow-up outpatient visits aimed at assessing compliance to therapy, home mechanical ventilation and/or LTOT **Duration:** 12 months **Follow-up**: 12 months	**Adherence**: N/D **Satisfaction**: N/D **Acceptability:** N/D
Walters et al., 2013 (53) *Internal Medicine J* Australia RCT *National Health and Medical Research Council, a Royal Hobart Hospital Research Foundation and a University of Tasmania Institutional Research* funded	**Sample size:** 90 **Mean age/age range:** 68.2 **Gender ratio (M/F):** 49M/41F **Pulmonary disease:** diagnosis of COPD, COPD hospitalizations (in the last year): n.5 **Comorbidities:** N/D, mean 1.6 **Smoking:** n. 43 (current smokers) **Ongoing pharmacological therapy:** Inhaled steroids: n.29; Oral steroids: n.2; Bronchodilators (short-acting): n.27; Bronchodilators (long-acting): n. 42; Medical Research Council dyspnoea score: 2.8	**Type of intervention in telenursing**: Telephone health mentoring program by community nurses trained in a behavioral approach (goal setting, action planning, problem solving, motivation), based on the SNAPPS model (smoking, nutrition, alcohol, physical activity, psychosocial well-being, symptom management). **Digital tool used:** telephone **Duration:** 12 months **Follow-up:** 6 and 12 months **Other type of intervention**: N/D **Duration**: N/D **Follow-up**: N/D	**Adherence**: the median number of telephone contacts was 9.5 (range 1–21) with a duration of approximately 30 min **Satisfaction**: evaluated through Satisfaction With Life Scale. At baseline 23.9, at 6 months 24.8 (n.74 patients), at 12 months 24.4 (n.74 patients) **Acceptability:** good (demonstrated by the high number of patients who participated in telephone calls) but high drop-out rate (12 withdrawals)
	**Sample size:** 92 **Mean age/age range:** 67.3 **Gender ratio (M/F):** 47M/45F **Pulmonary disease:** diagnosis of COPD, COPD hospitalizations (in the last year): n.0 **Comorbidities:** N/D, mean 1.7 **Smoking:** n. 33 (current smokers) **Ongoing pharmacological therapy:** inhaled steroids: n.48; oral steroids: n.5; bronchodilators (short-acting): n.47; bronchodilators (long-acting): n. 69; Medical Research Council dyspnea score: 2.5	**Type of intervention**: Usual care+monthly non-interventional phone calls **Duration:** 12 months **Follow-up:** 6 and 12 months	**Adherence**: the median number of telephone contacts was 9 (range 1–14) but of short duration (about 1 min) **Satisfaction**: evaluated through the Satisfaction With Life Scale. At baseline 22.0, at 6 months 23.5 (n.83 patients), at 12 months 23.4 (n.80 patients) **Acceptability:** N/D
Wong et al., 2005 (56) *J Adv Nurs* Hong Kong RCT No Funding	**Sample size:** 30 **Mean age/age range:** 72.8 **Gender ratio (M/F):** 27M/3F **Pulmonary disease:** diagnosis of COPD **Comorbidities:** N/D **Smoking:** n.5 (current smoker), n.25 (former or never smoker) **Ongoing pharmacological therapy:** O_2_ therapy: n. 3 patients	**Type of intervention in telenursing**: post-discharge telephone follow-up with two structured and personalized telephone calls **Digital tool used:** telephone with recorder **Duration:** 3 months **Follow-up:** two phone calls (day 3–7 and day 14–20 post-discharge) **Other type of intervention**: N/D **Duration**: N/D **Follow-up**: N/D	**Adherence**: not evaluated with a validated method but high (patients responded and participated in the calls. The intervention also promoted adherence to home pharmacological therapy, thanks to the positive reinforcement. **Satisfaction**: N/D **Acceptability:** N/D
	**Sample size:** 30 **Mean age/age range:** 74.4 **Gender ratio (M/F):** 20M/10F **Pulmonary disease:** diagnosis of COPD **Comorbidities:** NR **Smoking:** n.5 (current smoker), n.25 (former or never smoker) **Ongoing pharmacological therapy:** O_2_ therapy: n. 6 patients	**Type of intervention**: normal routine care without telephone follow-up **Duration:** 3 months **Follow-up:** N/D	**Adherence**: N/D **Satisfaction**: N/D **Acceptability:** N/D

Comparison groups are highlighted in light grey.

Randomized Controlled Trial, “RCT”; Missing Data, “MD”; Not Defined, “N/D”; Male, “M”; Female, “F”; percentage, “%”; Chronic Obstructive Pulmonary Disease, “COPD”; Global Initiative for Chronic Obstructive Lung Disease, “GOLD”; Emergency Department, “ED”; Oxygen, “O_2_”; Peripheral capillary oxygen saturation, “SpO_2_”; Forced Expiratory Volume in one second, “FEV_1_”; Length of Stay, “LOS”; Home Oxygen Therapy, “HOT”; Non-Invasive Ventilation, “NIV”; Long Term Oxygen Treatment, “LTOT”; Telemedicine Video Consultation, “TVC”; Medicines Adherence Report Scale, “MARS”; Usability, Satisfaction and Ease of Use Questionnaire, “USE-Q”.

#### 3.2.1 Telenursing: population

The total sample of patients who received telenursing interventions included in all studies was 2,731 patients with chronic respiratory diseases. The smaller study recruited 7 participants (34), while the largest study enrolled 530 participants (36). The mean sample size analysed from all studies was 65.02 patients.

Most of the included studies focused on COPD populations with a total population of 2,163 patients with COPD (21–33, 36, 38, 40–45, 47–58).

Seven studies investigated asthma populations with a total sample of 465 patients (20, 29, 34, 35, 37, 39, 46).

One study considered also patients with chronic respiratory failure (33 patients) (23), another also analysed patients with chronic respiratory failure associated with restrictive lung disease (14 patients), neuromuscular disease (24 patients), amyotrophic lateral sclerosis (12 patients) and other diseases non specified (11 patients) (47), and another study analysed patients with pulmonary cell hypoventilation syndrome (5 patients), pulmonary tuberculosis sequelae (2 patients) and other disease not specified (2 patients) (44).

The mean age of participants was reported in thirty-seven (15–23, 25–52) of thirty-nine studies and was 64.8 ± 1.5 years in a sample of 2,655 patients.

The COPD patients were consistently older, with a mean age of 70.5 ± 1.7 years in a sample of 2,032 patients (21, 22, 24–29, 31–33, 36, 38, 40–43, 45, 48, 49, 51–55, 58).

The asthma patient cohorts included younger populations, with a mean age of 38.1 years in a sample of 418 patients (20, 29, 34, 35, 37, 39, 46).

Gender distribution was calculated by thirty-seven studies (14–52) for a total of 1,427 males (52.2%) and 1,228 females (47.8%). When stratified by respiratory disease, COPD studies included more males (1,139 vs. 846), whereas asthma studies included more females (175 vs. 290).

COPD populations tended to be male-dominated (1,139 males vs. 846 females) (21–24, 26–29, 31–33, 36, 38, 40–45, 47–57).

Asthma studies showed a predominance of female patients (290 females vs. 175 males) (20, 29, 34, 35, 37, 39, 46).

Twenty-seven studies (20, 22, 27–30, 32, 33, 35–41, 44–53, 55–57) assessed smoking history for a total of 1,713 patients. One study calculated this parameter by referring to the number of packs per year (58 ± 43 packs for 53 patients/year) (49). The other studies (20, 22, 27–30, 32, 33, 35–41, 44–48, 50–53, 55–57) defined patients as current smokers, former smokers, or nonsmokers. Current smokers numbered 410 patients [out of a sample of 1,631 in twenty-six studies (20, 22, 27–30, 32, 33, 35–41, 44–48, 50–53, 56, 57)], former smokers numbered 692 patients [out of a sample of 1,139 in seventeen studies (20, 22, 28–30, 37, 40, 41, 44, 46, 47, 50–52, 55, 57)], and nonsmokers numbered 108 (out of a sample of 1,139 in nine studies (22, 29, 30, 37, 46, 48, 50–52).

Smoking history, reported in 27 studies (20, 22, 27–30, 32, 33, 35–41, 44–53, 55–57) (1,713 patients) showed a higher proportion of current and former smokers among COPD patients compared to asthma patients.

Smoking history for the COPD patient subpopulation was calculated in twenty studies with a total of 1,069 patients divided as current (309 out of 1,040 patients analysed in nineteen studies), former (560 out of 790 patients analysed in twelve studies), and nonsmokers (16 out of 339 patients analysed in seven studies). Smoking history for the asthma patient subpopulation was calculated in six studies with a total of 458 patients divided as current (94 out of 458 patients analysed in six studies), former (67 out of 216 patients analysed in four studies), and nonsmokers (92 out of 169 patients analysed in three studies).

The comorbidities reported were: heart failure (116 patients) (23, 40, 49, 52, 54), cardiovascular disease non specified (100 patients) (29, 40, 41), depression (73 patients) (40, 41, 52), diabetes (64 patients) (23, 29, 49, 52, 54), infection (59 patients) (52), hypertension (54 patients) (23, 49), osteoporosis (47 patients) (40, 52), myocardial infraction (35 patients) (23, 54), mental illness (22 patients) (34, 35), cerebrovascular disease (13 patients) (23, 39), coronary artery disease (12 patients) (49), gastrointestinal disease non specified (11 patients) (29), substance use disorder (8 patients) (34, 35), pulmonary hypertension (4 patients) (23), and angina (1 patients) (23).

Nine studies (28, 31–33, 38–51) described high proportions of participants with comorbidities without specifying them.

Twenty-three studies (21, 23, 27, 30, 34, 36–38, 40–42, 46, 47, 49, 50, 52–57) reported that participants continued their usual pharmacological treatment during the intervention period. Ongoing therapy included long term oxygen treatment (LTOT) (717 patients) (23, 30, 36, 41, 42, 45, 47, 50, 52), non-invasive ventilation (NIV) (639 patients) (23, 40–42, 44, 47), home oxygen therapy (HOT) (92 patients) (21, 27, 38, 44, 54, 56); the most frequently reported drugs therapies were inhaled medications, including long-acting bronchodilators (240 patients) (21, 37, 46, 49, 53, 57), corticosteroids (unspecified route of administration) (145 patients) (21, 41), short-acting (135 patients) (21, 49, 53, 57), bronchodilator (unspecified route of administration) (99 patients) (41), antibiotics (95 patients) (41, 57), inhaled (84 patients) (37, 49, 53, 57), long-acting anticholinergic (36 patients) (49), mucolytics (11 patients) (55), oral corticosteroids (8 patients) (53, 55, 57), and theophylline (2 patients on a sample of 29) (55).

#### 3.2.2 Telenursing: intervention

Twenty-seven studies (20, 23, 25, 26, 28, 30–38, 41–43, 45, 47–52, 55, 57) used telemonitoring systems that allowed patients to daily or near-daily record and transmit physiological parameters such as oxygen saturation, respiratory rate, heart rate, blood pressure, temperature, weight, and spirometry data. These interventions relied on digital platforms or handheld devices connected via telephone or internet to hospital-based nursing teams. Nurses reviewed the transmitted data daily, responding to alerts when thresholds were exceeded and providing feedback or referrals as needed. Shorter post-discharge telemonitoring programs were reported by four studies (40, 41, 45, 52), focusing on early detection of exacerbations and prevention of hospital readmissions. Other telemonitoring programs included structured alert systems (23, 36) and interactive screens for symptom tracking (57).

Nine studies (21, 22, 29, 40, 44, 46, 53, 54, 56) primarily used telephone-based interventions, in which nurses provided structured follow-up, educational reinforcement, or psychological support. Post-discharge telephone support models were described in two studies (29, 40), which combined clinical assessment with motivational coaching. Another study (39) used SMS and email reminders as an extension of telephone-based nursing communication to reinforce adherence and self-management.

Six studies (30, 34, 40, 41, 45, 52) integrated video-based consultations or synchronous telecommunication between patients and nurses, where patients engaged in video consultations following hospital discharge or during rehabilitation (58).

Seven studies (34, 35, 37, 39, 40, 46, 54) employed mobile health (mHealth) solutions to manage asthma and COPD through smartphone apps, SMS systems, or digital diaries; patients recorded peak expiratory flow and symptoms via mobile applications, receiving automated feedback or nurse-delivered coaching. These interventions focused primarily on enhancing self-management and adherence to inhaler therapy, often incorporating educational reminders and individualized action plans.

Six studies described multicomponent or hybrid interventions combining telemonitoring with tele-rehabilitation, education, or multidisciplinary care. Two studies (47, 58) integrated physical exercise programs, patient education, and remote supervision into their telemonitoring systems. Similarly, three studies (30, 36, 50) employed combined hospital-home models with nursing oversight.

#### 3.2.3 Telenursing: patient-reported outcomes

Adherence was variably assessed across the fourteen included studies (24, 28, 29, 31, 32, 34, 35, 37, 47, 48, 51, 53, 55, 56). Among the studies providing measurable results, adherence to telemonitoring or self-reporting activities ranged from 40% (48) to 97% (32), depending on the monitored parameter and the technology used. One study (48), reported the median adherence to daily physiological measurements (80%), with the highest rates observed for blood pressure (83%) and oxygen saturation (81%).

Another study (35) reported an increase in adherence from 45.58% to 85.29% over time, while two studies (28, 51) using the Medicines Adherence Report Scale (MARS) found stable or slightly improved scores during the intervention period.

A high level of adherence was also reflected by consistent data transmission rates (up to 88.9%) and regular participation in telemonitoring sessions (31).

Patient satisfaction was evaluated in nineteen studies (22, 24–26, 30, 32, 34, 36, 38–42, 45, 48, 50, 53, 55, 58) predominantly through non-validated questionnaires or interviews. Reported satisfaction rates ranged between 77% (36) and 94% (48), and a mean satisfaction score above 6/7 using the User Satisfaction Evaluation Questionnaire (USE-Q) scale was reported in another study (34).

Patients described telemonitoring systems as useful, easy to use, and helpful in managing their chronic respiratory disease; participants also expressed an enhanced sense of control and confidence in self-management, and a perception of improved communication with healthcare professionals (22, 25, 26, 30, 32, 38–42, 45, 50, 53, 55, 58). One study reported that 91% of participants were satisfied with the telecare service, and 100% with the nursing support provided (24).

Patient acceptability was evaluated in five studies (22, 34, 35, 48, 53). Participants described the systems as useful, non-invasive, and easy to integrate into daily routines (22, 34, 53).

Reported acceptability rates were 93.9% (48) and 95.7% (35).

One study noted attrition and dropout during follow-up, indicating that sustained engagement was influenced by the frequency and complexity of monitoring tasks (22).

### 3.3 Traditional care

Table 1 summarizes the studies and population characteristics, the characteristics of interventions performed through traditional care, and the related patients' reported outcomes (comparison group).

#### 3.3.1 Traditional care: population

Twenty-eight studies included in this systematic review (20–22, 24, 26, 28–30, 32, 33, 36–40, 44–49, 51–53, 55–58) reported a direct comparison between the telenursing care (intervention) and traditional care (comparison).

The total sample of patients who received traditional care included in all studies was 1,609 patients with chronic respiratory diseases.

The smaller study recruited 12 participants (29), while the largest study enrolled 212 participants (39). The mean sample size analyzed from all comparison studies was 57.5 patients.

Most of the included studies (22 studies (21, 22, 24, 26, 28–30, 32, 33, 36, 38, 40, 45, 48, 49, 51–53, 55–58) focused on COPD populations with a total population of 1,068 patients.

Five studies investigated asthma populations (total sample of 423 patients) (20, 29, 37, 39, 46).

One study (47) analyzed also patients with chronic respiratory failure associated with neuromuscular disease (26 patients), restrictive lung disease (14 patients), amyotrophic lateral sclerosis (10 patients) and other diseases non specified (8 patients); another study (44) analyzed patients with pulmonary cell hypoventilation syndrome (5 patients) and pulmonary tuberculosis sequelae (4 patients).

The mean age of participants was reported in thirty-six of twenty-eight studies (21, 22, 24, 26, 28, 29, 32, 33, 36–40, 44–49, 51–53, 55–58) and was 63.2 years in a sample of 1,530 patients.

The COPD patient cohorts were older, with a mean age of 70.9 years in a sample of 1,040 patients (21, 22, 24, 26, 28, 29, 32, 33, 36, 38, 40, 44–49, 51–53, 55–58).

The asthma patient cohorts included younger populations, with a mean age of 42 years in a sample of 372 patients (29, 37, 39, 46).

Gender distribution was calculated by twenty-seven studies (20–22, 24, 26, 28–30, 32, 33, 36–40, 44–49, 51–53, 55–57) for a total of 790 males and 770 females.

COPD populations tended to be male-dominated (541 males vs. 527 females in twenty-one studies) (21, 22, 24, 26, 28–30, 32, 33, 36, 38, 40, 45, 48, 49, 51–53, 55–57).

Asthma studies showed a predominance of female patients (256 females vs. 167 males in five studies) (20, 29, 37, 39, 46).

Twenty-three studies assessed smoking history for a total of 1,399 patients (20, 22, 28–30, 32, 33, 36, 37, 39, 40, 44–49, 51–53, 55–57). One study calculated this parameter by referring to the number of packs per year (47 ± 31 packs for 134 patients/year) (49). The other studies (20, 22, 28–30, 32, 33, 36, 37, 39, 40, 44–48, 51–53, 55–57) defined patients as current smokers, former smokers, or nonsmokers. Current smokers numbered 334 patients (out of a sample of 1,316 in twenty-one studies (20, 22, 28–30, 32, 33, 36, 37, 39, 40, 44–48, 51–53, 56, 57), former smokers numbered 507 patients (out of a sample of 872 in fourteen studies (20, 22, 28–30, 37, 40, 44, 46, 47, 51, 52, 55, 57), and nonsmokers numbered 92 patients (out of a sample of 526 in eight studies (22, 29, 30, 37, 46, 48, 51, 52).

Smoking history for the COPD patient subpopulation was calculated in seventeen studies with a total of 805 patients divided as current (228 out of 775 patients analyzed in sixteen studies), former (391 out of 790 patients analyzed in ten studies), and nonsmokers (13 out of 366 patients analyzed in six studies). Smoking history for the asthma patient subpopulation was calculated in five studies (20, 29, 37, 39, 46) with a total of 423 patients divided as current [96 out of 423 patients analyzed in five studies (20, 29, 37, 39, 46)], former [73 out of 211 patients analyzed in four studies (20, 29, 37, 46)], and nonsmokers [79 out of 160 patients analyzed in three studies (29, 37, 46)].

The comorbidities reported were: infection (74 patients) (52), heart failure (54 patients) (40, 52), osteoporosis (40 patients) (40, 52), cardiovascular disease non specified (39 patients) (29, 40), diabetes (21 patients) (29, 52), depression (19 patients) (40, 52), cerebrovascular disease (11 patients) (52), gastrointestinal disease non specified (8 patients) (29).

Seven studies (28, 32, 33, 38, 39, 50, 51) described high proportions of participants with comorbidities without specifying them.

Sixteen studies (21, 30, 36–38, 40, 44–47, 49, 52, 53, 55–57) reported that participants continued their usual pharmacological treatment during the intervention period. Ongoing therapy included LTOT (108 patients) (30, 36, 40, 45, 47, 52), NIV (62 patients) (40, 44, 47), and HOT (56 patients) (21, 38, 44, 56); the most frequently reported drugs therapies were inhaled medications, including long-acting bronchodilators (236 patients) (21, 37, 46, 49, 53, 57), short-acting (157 patients) (21, 49, 53, 57), inhaled (111 patients) (37, 49, 53, 57), corticosteroids (unspecified route of administration) (45 patients) (21), oral corticosteroids (13 patients) (53, 55, 57), long acting anticholinergic (34 patients) (49), mucolytics (12 patients) (55), theophylline (3 patients) (55), and antibiotics (2 patients) (57).

#### 3.3.2 Traditional care: intervention

Across the studies included in this review, traditional care for patients with chronic respiratory diseases consisted of routine medical follow-up and pharmacological treatment provided by general practitioners or respiratory specialists, without the integration of telemonitoring, remote data transmission, or continuous nursing support (20–22, 24, 26, 28–30, 32, 33, 36–40, 44–49, 51–53, 55–58).

Four RCTs (32, 33, 47, 58) defined standard care as regular outpatient visits and conventional follow-up focused on symptom control, adherence to prescribed medication, and education about self-management of exacerbations, with no remote supervision. Three studies (30, 49, 52) described control groups receiving usual post-discharge follow-up, with scheduled in-person evaluations at specific intervals and telephone contact only when clinically necessary.

In one study (51), traditional care was complemented by structured education on self-management of exacerbations, reinforced by a written action plan and an emergency supply of antibiotics and steroids. Other studies (36, 38) defined the traditional home care without telemonitoring, following the hospital's usual COPD management protocols and reporting explicit baseline data on hospital admissions and emergency visits.

The other three studies (22, 24, 48) characterized the control condition as routine disease management through periodic clinical visits and education on self-care practices.

#### 3.3.3 Traditional care: patient-reported outcomes

Adherence was variably evaluated in four studies (29, 46, 51, 53), with generally stable results over time. One study (29) assessed adherence using the Adherence Starts with Knowledge questionnaire, reporting scores increasing from 17.0 to 17.5 in four patients and from 18.0 to 17.0 in five patients. One study (51) measured adherence through the MARS, showing minimal variation from 23.6 at baseline to 23.7 at 12 months in a sample of 101 patients. Another study (46) detected self-reported adherence increasing slightly from 6.19 (*n* *=* 91) to 6.37 (*n* *=* 92). One study (53) quantified adherence through the frequency of telephone contacts, reporting a median of nine contacts per patient (range 1–14), each lasting approximately one minute.

Patient satisfaction was reported in one study (53), which used the Satisfaction With Life Scale, observing a mean increase from 22.0 at baseline to 23.5 at six months and 23.4 at twelve months (*n* *=* 83 and *n* *=* 80, respectively).

No study provided quantitative measures of patient acceptability.

### 3.4 Telenursing vs. traditional care: outcomes

#### 3.4.1 Exacerbation rate

The exacerbation rate was evaluated in 11 studies (21, 27–29, 31, 36, 37, 47, 49, 50, 57) at different time points, as shown in Table 2.

**Table 2:** Mean and median of exacerbation rate at different time points, reported in each study for the telenursing group (intervention) and traditional care (comparison).

Study	12 months prior	Baseline	3 months	6 months	12 months
Telenursing (Intervention) Berkof et al. (21) Early et al. (27) Farmer et al. (28) Fox et al. (29) Kamei et al. (31) Mirón Rubio et al. (36) Nemanic et al. (37) Vitacca et al. (47) Te-Wei Ho et al. (49) Persson et al. (50) Trappenburg et al. (57)		0.30 (n.53), 0.41 (n.24), 0.94 (n.51), 1.00 (n.59), 1.43 (n.16), 2.17 (n.17), 3.7 (n. 11), 7 (n.8)	0.29 (n.24)	0.57 (n.16), 0.65 (n.59), 1.05 (n.17), 2.88 (n.25)	0.49 (n.51), 2.76 (n.97), 3.2 (n.20),
Total mean events (n. of patients evaluated)		1.2 (n. 239)	0.29 (n.24)	1.2 (n.117)	2.1 (n. 168)
Traditional care (Comparison) Berkof et al. (21) Farmer et al. (28) Fox et al. (29) Mirón Rubio et al. (36) Nemanic et al. (37) Vitacca et al. (47) Trappenburg et al. (57)	2.10 (n.28)	0.5 (n.12), 0.69 (n.56), 0.71 (n.49),1.31 (n.16),		0.25 (n.12), 0.87 (n.16), 1.01 (n.56)	0.48 (n.49), 9.36 (73)
Total mean events (n. of patients evaluated)	2.10 (n.28)	0.8 (n.133)		0.9 (n.84)	5.8 (n. 122)

For each value, the mean of exacerbation rate presentations reported in each study on the number of patients evaluated (n.) was specified at each time point.

#### 3.4.2 Emergency department (ED) presentations

The ED presentations were evaluated in 16 studies (20, 24, 26, 29, 31, 33, 34, 36, 38, 39, 43, 47, 49, 54–56) at different time points, as shown in Table 3.

**Table 3:** Mean and median of ED presentations at different time points, reported in each study for the telenursing group (intervention) and traditional care (comparison).

Study	12 months prior	Baseline	5 weeks	2 months	3 months	6 months	7 months	9 months	12 months
Telenursing (Intervention) Ahmed et al. (20) Park-Chun Chau et al. (24) De San Miguel et al. (26) Fox et al. (29) Kamei et al. (31) Lewis et al. (33) Mammen et al. (34) Mirón Rubio et al. (36) Paré et al. (38) Prabhakaran et al. (39) Shany et al. (43) Vitacca et al. (47) Te-Wei Ho et al. (49) Segrelles Calvo et al. (55) Wong et al. (56)		Mean: 0 (n.37), 0.06 (n.16), 0.15 (n.59), 0.17 (n.34), 0.28 (n.7), 0.35 (n.53), 2.01 (n.60), 3.9 (n.21), > 2 (n.2)	Mean: 0.08 (n.212)	Mean: 0 (n.15), 1–2 (n.7)	Mean: 0.11 (n.159), 0.1 (n.28)	Mean: 0.11 (n.17), 0 (n.17), 0.16 (n.36), 0.22 (n.53),0.33 (n.30), 0.58 (n.60), 1.26 (n.26)	0.68 (n.29)	0.71 (n.60)	Mean: 0.84 (n.97), 2.8 (n.21),
Total average mean events (n. of patients evaluated)		0.87 (n.287), ≥ 2 (n.2)	0.08 (n.212)	0 (n.15), 1–2 (n.7)	0.11 (n.187)	0.37 (n.239)	0.68 (n.29)	0.7 (n.60)	1.2 (n.118)
Traditional care (Comparison) Park-Chun Chau et al. (24) De San Miguel et al. (26) Fox et al. (29) Lewis et al. (33) Mirón Rubio et al. (36) Paré et al. (38) Prabhakaran et al. (39) Vitacca et al. (47) Te-Wei Ho et al. (49) Segrelles Calvo et al. (55)	Mean: 1.89 (n.28)	Mean: 0 (n.28), 0.02 (n.56), 0.32 (n.53), 2.11 (n.60)	Mean: 0.09 (n.170)	Mean: 0 (n.15), 1–2 (n.3)	Mean: 0.12 (n.162)	Mean: 0 (n.28), 0.31 (n.35), 0.54 (n.53), 0.63 (n.60)	Mean: 2.19 (n.26)	Mean: 1.21 (n.60)	Mean: 1.2 (n.73)
Total average mean events (n. of patients evaluated)	1.89 (n.28)	0.69 (n.197)	0.09 (n.170)	0 (n.15), 1–2 (n.3)	0.12 (n.162)	0.42 (n.176)	2.19 (n.26)	1.21 (n.60)	1.2 (n.73)

For each value, the mean of ED presentations reported in each study on the number of patients evaluated (n.) was specified at each time point.

#### 3.4.3 Hospitalization rate

The hospitalization rate was evaluated in 33 studies (20, 21, 23, 33, 36, 38–41, 43–45, 47–58) at different time points, as shown in Table 4.

**Table 4:** Mean and median of hospitalization rate at different time points, reported in each study for the telenursing group (intervention) and traditional care (comparison).

Studies	12 months prior	Baseline	1 month	5 weeks	2 months	3 months	4 months	6 months	7 months	9 months	10 months	12 months	26 months
Telenursing (Intervention) Ahmed et al. (20) Berkof et al. (21) Chatwin et al. (23) Park-Chun Chau et al. (24) Cooper et al. (25) De San Miguel et al. (26) Early et al. (27) Farmer et al. (28) Fox et al. (29) Kamei et al. (31) Lewis et al. (32) Mirón Rubio et al. (36) Paré et al. (38) Prabhakaran et al. (39) Saleh et al. (40) Saleh et al. (41) Shany et al. (43) Shimoyama et al. (44) Sorknaes et al. (45) Vitacca et al. (47) Antoniades et al. (48) Te-Wei Ho et al. (49) Persson et al. (50) Pinnock et al. (51) Sorknaes et al. (52) Walters et al. (53) Roberts et al. (54) Segrelles Calvo et al. (55) Wong et al. (56) Trappenburg (57) Dinesen et al. (58)		Mean: 0 (n.110), 0.05 (n.107), 0.16 (n.6), 0.17 (n.17), 0.42 (n.59), 0.44 (n.52), 0.45 (n.57), 1.0 (n.119), 1.03 (n.29), 1.46 (n.60), 2.27 (n.132), 2.41 (n.22), 3.04 (n.21), > 1 (n.6), > 2 (n.10), 1 or 2 (n.530)	Mean: 0.12 (n.50), 0.36 (n.130)	Mean: 0.04 (n.212)	Mean: 0 (n.15), 0.63 (n.127), 1–2 (n.7)	Mean: 0.07 (n.159), 0.20 (n.24), 0.6 (n.30), 0.77 (n.127)	Mean: 0.22 (n.9), 0.5 (n.10)	Mean: 0 (n.122), 0.07 (n.27), 0.18 (n.53), 0.22 (n.36), 0.23 (n.17), 0.41 (n.60), 0.47 (n.57), 0.5 (n.26), 1.22 (n.121), > 2 (n.43)	Mean:0.41 (n.29)	Mean: 0.38 (n.60)	Mean: 0.49 (n. 54)	Mean: 0 (n.61), 0.06 (n.15), 0.07 (n.14), 0.1 (n.10), 0.14 (n.173), 0.39 (n.96), 0.6 (n.20), 1.3 (n.16), 1.5 (n.97), 2.38 (n.21), > 2 (n.64)	Mean: 0.18 (n.118)
Total mean events (n. of patients evaluated)		0.95 (n.791), > 1 (n.6), ≥ 2 (n.10), 1 or 2 (n.530)	0.3 (n.180)	0.04 (n.212)	0.56 (n.142), 1–2 (n.7)	0.38 (n. 340)	0.37 (n.19)	0.46 (n.519), ≥ 2 (n.43)	0.41 (n.29)	0.38 (n.60)	0.49 (n.54)	0.56 (n.523), ≥ 2 (n.64)	0.18 (n.118)
Traditional care (Comparison) Ahmed et al. (20) Berkof et al. (21) Park-Chun Chau et al. (24) De San Miguel et al. (26) Fox et al. (29) Jakobsen et al. (30) Lewis et al. (32) Mirón Rubio et al. (36) Paré et al. (38) Prabhakaran et al. (39) Saleh et al. (40) Shimoyama et al. (44) Sorknaes et al. (45) Vitacca et al. (47) Antoniades et al. (48) Te-Wei Ho et al. (49) Pinnock et al. (51) Sorknaes et al. (52) Walters et al. (53) Segrelles Calvo et al. (55) Wong et al. (56) Trappenburg (57) Dinesen et al. (58)	Mean: 1.17 (n.28)	Mean: 0 (n.203), 0.06 (n.16), 0.14 (n.7), 0.16 (n.6), 0.34 (n.49), 0.35 (n.57), 0.36 (n.53), 0.96 (n.30), 1.0 (n.1), 1.73 (n.60), 2.64 (n.134), 2.89 (n.18), > 1 (n.4), > 2 (n.4)	Mean: 0.22 (n.50), 0.28 (n.131)	Mean: 0.04 (n.170)	Mean: 0 (n.15), 0.47 (n.130), 1–2 (n.3),	Mean: 0.05 (n.162), 0.72 (n.126)		Mean: 0 (n.61), 0.35 (n.20), 0.47 (n.60), 0.49 (n.88), 1.0 (n.12), 1.28 (n.121), > 2 (n.11)	Mean: 1.26 (n.26)	Mean: 0.61 (n.60)	Mean: 1.17 (n.45)	Mean: 0.06 (n.80), 1.1 (n.106), 1.34 (n.49), 1.5 (n.20), 2.64 (n.73)	
Total mean events (n. of patients evaluated)	1.17 (n.28)	0.95 (n. 634)	0.3 (n.181)	0.04 (n.170)	0.42 (n.145), 1–2 (n.3),	0.34 (n.288)		0.67 (n.362), > 2 (n.11)	1.26 (n.26)	0.61 (n.60)	1.17 (n.45)	1.25 (n.328)	

For each value, the mean of hospitalization rate presentations reported in each study on the number of patients evaluated (n.) was specified at each time point.

#### 3.4.4 Hospital length of stay (LOS)

The hospital length of stay (LOS) was evaluated in 16 studies (21, 24, 26, 30, 32, 36, 38, 40, 43–45, 48, 51, 52, 55, 57) at different time points, as shown in Table 5.

**Table 5:** Mean and median of LOS in days at different time points, reported in each study for the telenursing group (intervention) and traditional care (comparison).

Studies	12 months prior	Baseline	1 month	2 months	3 months	6 months	7 months	9 months	12 months
Telenursing (Intervention) ** (**21, 24, 26, 32, 36, 38, 40, 43–45, 48, 51, 52, 55, 57**)**		Mean: 7.66 (n.22), 9 (n.21), 9.1 (n.59), 15.1 (n.60)	Mean: 0.3 (n.50), 1.02 (n.130)	Mean 2.16 (n.22), 2.34 (n.127)	Mean: 2.77 (n.127) Median: 0 (n.15)	Mean: 2.4 (n.36), 4.5 (n.96), 4.95 (n.121), 6.5 (n.60), 6.6 (n.59), 6.84 (n.26)	Mean: 3.62 (n.29)	Mean: 4.3 (n.60)	Mean: 6.3 (n.56), 7.02 (n.57), 8.29 (n.59), 9.5 (n.128),11.4 (n.16), 20.6 (n.21)
Total mean in days (n. of patients evaluated		11.1 (n.162)	0.8 (n.180)	2.3 (n.149)	2.77 (n.127)	5.2 (n.398)	3.62 (n.29)	4.3 (n.60)	9.1 (n.337)
Traditional care (Comparison) **(**24, 26, 30, 32, 36, 38, 40, 44, 45, 48, 51, 52, 55, 57**)**	Mean: 6.42 (n.28)	Mean: 1.5 (n.50), 6.56 (n.56), 8.06 (n.28), 14.05 (n.60)	Mean: 1.06 (n.50), 1.18 (n.131)	Mean 0.78 (n.18), 2.70 (n.130)	Mean: 3.93 (n.126)	Mean: 3.45 (n.60), 4.6 (n.35), 6.37 (n.121), 7.46 (n.56)	Mean: 10.6 (n.26)	Mean: 3.53 (n.60)	Mean: 7.46 (n.49), 8.8 (n.106), 15.6 (n.20)
Total mean in days (n. of patients evaluated	6.42 (n.28)	7.8 (n. 194)	1.1 (n.181)	2.5 (n.148)	3.93 (n.126)	5.7 (n.272)	10.6 (n.26)	3.53 (n.60)	9.2 (n.175)

For each value, the mean of LOS in days reported in each study on the number of patients evaluated (n.) was specified at each time point.

### 3.5 Risk of bias and overall quality

The risk of bias of the case series included in the present systematic review was assessed using the JBI for case series (19) and displayed in Supplementary Table S6.

**Figure 2:**
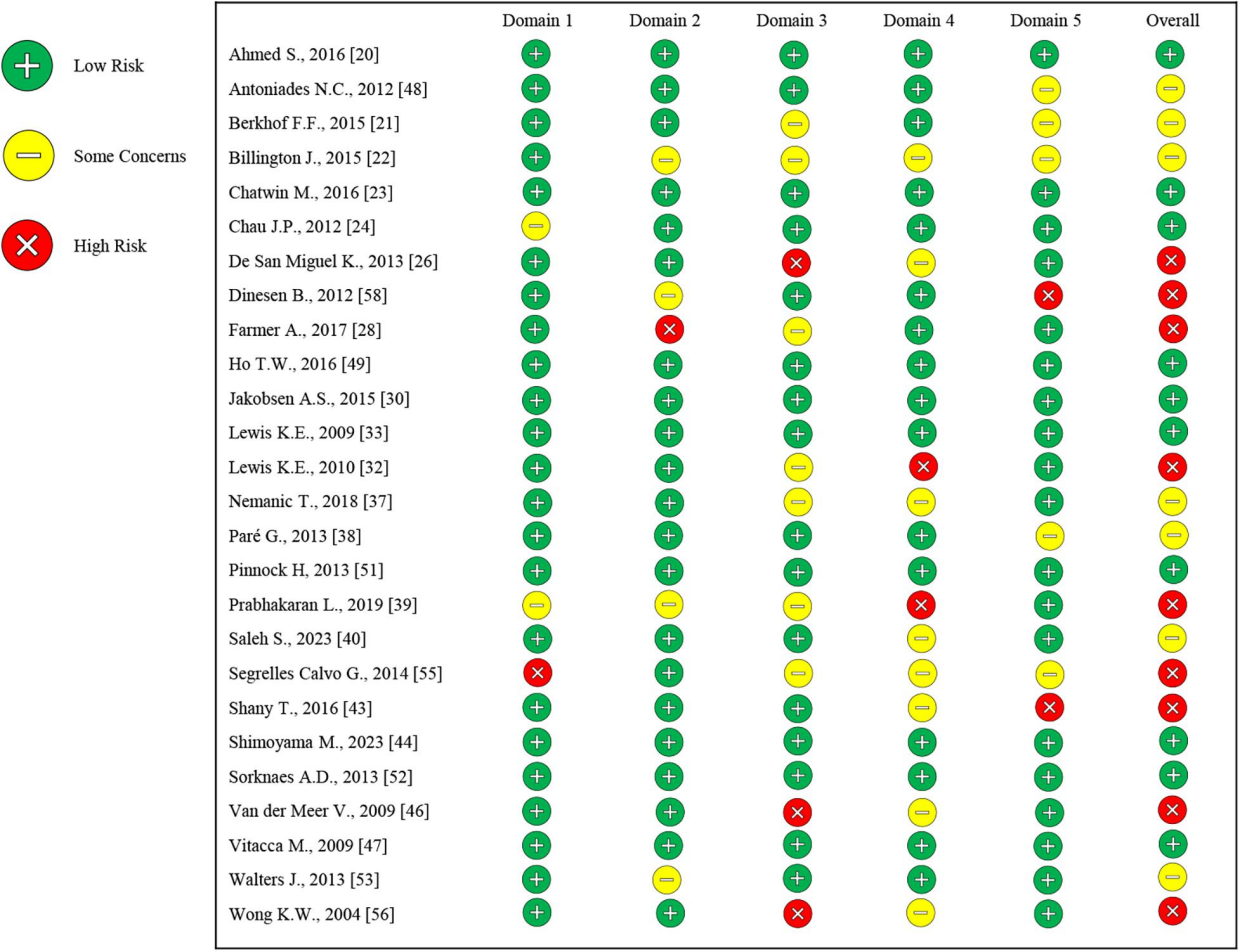
Risk of bias of the included non-RCTs.

The risk of bias of the non-RCTs included in the present systematic review was assessed using the ROBINS-I and displayed in Figure 2.

Three studies were judged with a serious risk of bias (25, 35, 41), six studies (29, 31, 36, 42, 54, 57) with a moderate risk, and three (34, 45, 50) with a low risk.

**Figure 3:**
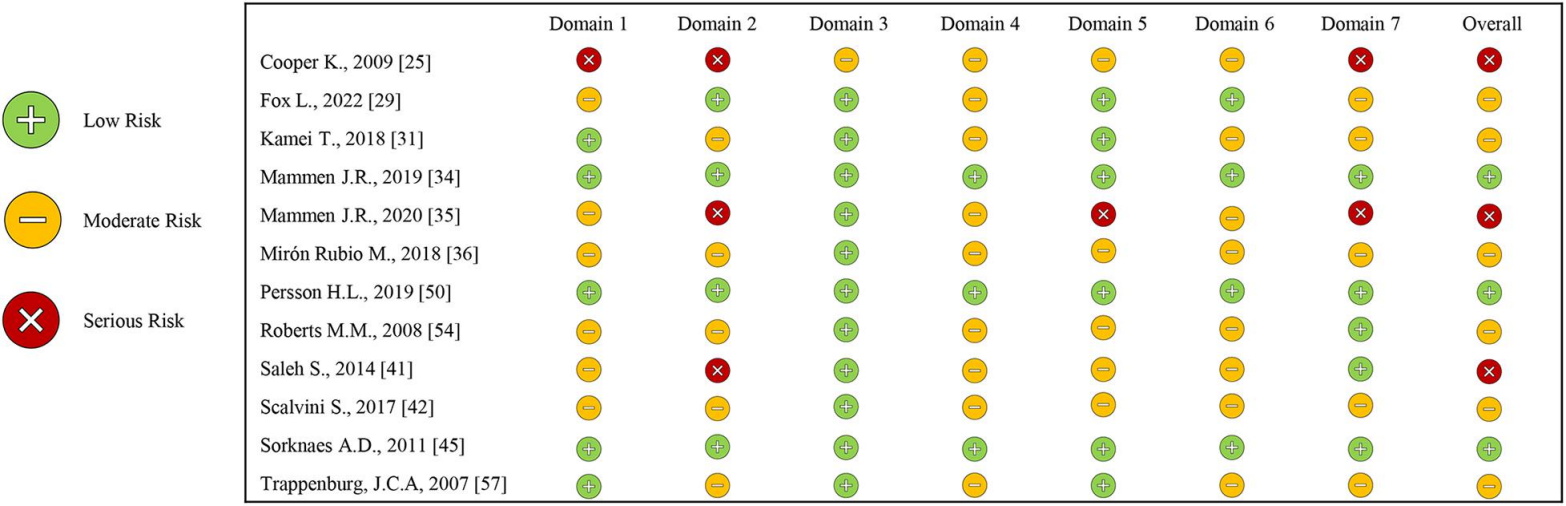
Risk of bias of the included RCTs.

The risk of bias of the RCTs included in the present systematic review was assessed using the RoB-2 and displayed in Figure 3.

Nine studies were judged with a high risk of bias (26, 28, 32, 39, 43, 46, 55, 56, 58), seven studies (21, 22, 37, 38, 40, 48, 53), had some concerns, and eleven (20, 23, 24, 30, 32, 33, 44, 47, 49, 51, 52) with a low risk.

## 4 Discussion

The present systematic review synthesized the findings of thirty-nine studies including 4,340 adult subjects (3,231 affected by COPD and 888 by asthma), to evaluate the impact of telenursing in chronic respiratory disease management on the health care systems compared to traditional care.

Telenursing represents a modern evolution of nursing care delivery, which ensures continuity of care and proactive management of patients with chronic diseases (10, 59). Previous studies highlighted that leveraging digital technologies, such as telephones, videoconferencing, mobile applications, and telemonitoring systems, health care services can be provided remotely with comparable efficacy and efficiency to traditional in-person care (10, 59, 60). Previous studies highlighted the clinical efficacy of telenursing in the management of chronic disease, extending beyond respiratory conditions, such as cardiovascular (61), metabolic (62), and oncological diseases (63). Across these contexts, the implementation of telenursing has been associated with improved outcomes, including reduced hospitalizations and ED presentations, improvements in quality of life and overall health status, enhanced self-management of chronic conditions, and reduced symptom-related distress (61–63). The present systematic reviews indicate that telenursing, delivered through structured nurse-guided telemonitoring, video consultations, or telephone follow-ups, was slightly effective in reducing hospital admissions and exacerbations, enhancing long-term treatment adherence, suggesting a potential role of telenursing in supporting healthcare systems.

Although the present research aims to cover telenursing in all chronic respiratory diseases (*n* *=* 2,731 patients included in telenursing group), the studies included a population of patients with COPD (*n* *=* 2,163) or asthma (*n* *=* 465), showing the possibility of expanding the frontiers of telenursing to include the management of other chronic respiratory diseases, such as cystic fibrosis or pulmonary hypertension in adult patients.

The pooled analysis of the thirty-nine included studies (20–58) revealed a heterogeneous population in terms of demographics between the patients affected by COPD or asthma, although consistent trends were observed in line with international literature.

The mean age of participants in the telenursing group was 64.8 ± 1.5 years, with higher values observed in the COPD subgroup (Mean: 70.5 ± 1.7 years) compared with the asthma subgroup (Mean: 38.1 years). This age difference reflects the distinct etiopathogenesis of the two diseases: COPD is closely linked to cumulative risk factors such as advanced age, chronic exposure to cigarette smoke, and environmental pollutants (3, 64), whereas asthma is distinguished in the more common early (<12 years of age) and late-onset asthma, which is more frequent in female smokers (65).

The observed gender disparity, especially across COPD patients, is consistent with existing literature; in fact, previous evidence showed that COPD is more common among males due to greater exposure to tobacco smoke and occupational pollutants (66, 67). Nonetheless, in recent decades, a progressive increase in COPD prevalence among females has been observed, likely attributable to changing smoking habits and greater susceptibility to airborne pollutants (68–70). Conversely, asthma is more prevalent among adult females (65), who often have a family or personal history of atopic status (71), partly due to hormonal and immunological factors (72–74).

The highest proportion of current and former smokers among COPD patients, confirming the strong etiological link between smoking and disease onset (75–77). In contrast, smoking prevalence among asthma patients was substantially lower, with a predominance of former or never smokers. Nonetheless, even among asthma patients, exposure to cigarette smoke, including second-hand smoke (78), remains a significant clinical risk factor, contributing to poorer disease control and reduced responsiveness to inhaled corticosteroids (79, 80).

Across studies, telenursing interventions were associated with a reduction in respiratory exacerbation rates. The number of respiratory exacerbations per patient was generally lower in the telenursing groups. At six months, the intervention group reported a mean of 1.2 exacerbations (*n* *=* 117 patients) compared with 0.9 in the control group (*n* *=* 176), while at twelve months, the means were 2.1 (*n* *=* 168) in the intervention group and 5.8 (*n* *=* 122) in the control group.

These findings suggest that early symptom detection through remote nursing surveillance and prompt intervention may contribute to a reduction in respiratory exacerbations, particularly in long-term follow-up (31).

Interestingly, the findings on the exacerbation rates in the present study were not substantially influenced by data from Japanese studies. In fact, only one study conducted in Japan by Kamei et al. (31) contributed to the exacerbation rate outcome. This aspect is relevant, as previous literature has reported lower COPD exacerbation rates in Japan compared with other countries (81). Such differences have been hypothesized to be related to contextual factors, including environmental conditions, lifestyle, and potentially greater access to healthcare services (81). However, current evidence remains insufficient to confirm or refute these explanations (81). Therefore, the limited contribution of Japanese data in the present review reduces the likelihood that geographical variability influenced the overall findings on exacerbation rates.

Despite the reduction in exacerbation rates, presentations to the Emergency Department (ED) remained stable across both intervention and control groups: at twelve months, the mean number of ED visits per patient was 1.2 in both the intervention (*n* *=* 118) and control groups (*n* *=* 73). The stability of ED visits despite the reduction in exacerbation rates suggests either that some acute presentations were not preventable by telenursing (e.g., sudden-onset events or comorbid emergencies) or that patients still preferred ED assessment despite effective remote management, potentially because of local care pathways or patient preference. These findings were partially in line with the results of the systematic review of Kim et al. (2,023), which showed a reduction in ED presentations in the telenursing group, even if in a non-statistically significant manner, in patients with colorectal cancer (82).

However, the reduction in exacerbation rates was reflected in lower hospital admission rates in the telenursing group compared with traditional care at both the six- and twelve-month follow-ups. At six months, the intervention group reported a mean of 0.46 hospitalizations (*n* *=* 519) compared with 0.67 in the control group (*n* *=* 362). At twelve months, the difference widened, with a mean of 0.56 hospitalizations in the intervention group (*n* *=* 523) vs. 1.25 in the control group (*n* *=* 328). No readmissions were also registered in either group, with zero hospitalizations observed among samples of 61 control and 122 intervention patients at six months, and among 61 patients in the control group at twelve months.

A deeper interpretation of these findings can be derived by taking into consideration the characteristics of the interventions implemented across the included studies. While traditional care mainly consisted of periodic in-person follow-up and pharmacological management without continuous monitoring or remote nursing support (20–22, 24, 26, 28–30, 32, 33, 36–40, 44–49, 51–53, 55–58), telenursing interventions introduced varying degrees of remote supervision, ranging from simple telephone follow-ups to complex telemonitoring systems and multicomponent digital platforms.

In particular, telemonitoring-based interventions, which represented the most frequently adopted approach in telenursing (20, 23, 25, 26, 28, 30–38, 41–43, 45, 47–52, 55, 57), enabled continuous or near-daily transmission of physiological and symptom data, allowing nurses to detect early signs of clinical deterioration and intervene promptly. This real-time monitoring may explain the observed reduction in exacerbations and hospital admissions, as it facilitates timely clinical decision-making and prevents the progression of acute events. The observed reduction in exacerbation rates and hospital admissions suggests the potential of telenursing to improve disease management and reduce acute events in patients with chronic respiratory conditions, enabling earlier recognition of symptom worsening and timely adjustment of treatment plans, thereby preventing progression to severe episodes (10, 59, 62).

In particular, these findings suggest that telenursing could be considered as a component of long-term disease management to reduce inpatient burden. In fact, the widening difference between groups at twelve months indicates that the benefits of telenursing are more evident with sustained monitoring and patient engagement over time. Consistent with previous reviews (62) on telehealth in chronic disease management, these results reinforce the importance of continuous telemedicine monitoring as a cost-effective strategy to optimize disease control and reduce hospital burden.

However, the stability in ED presentations despite the reduction in exacerbation rates also highlights the need to strengthen patient confidence in telenursing systems that have demonstrated effectiveness, and to enhance patient education, access to primary care, and triage protocols that could translate the reduction in exacerbations into fewer ED presentations. In fact, patient education and awareness on chronic disease self-management were recognized as critical components to reduce healthcare system costs and ED presentations (83). Moreover, telenursing interventions could incorporate structured educational modules and self-management coaching aimed at improving patients' ability to recognize early symptoms and make appropriate care decisions. Previous evidence showed that during the COVID-19 pandemic era, pre-hospitalization telephonic triage by nurses significantly reduced the risk of SARS-CoV-2 infection in both healthcare providers and surgical cancer patients, limiting the overload of the healthcare system (84–86).

**Figure 4:**
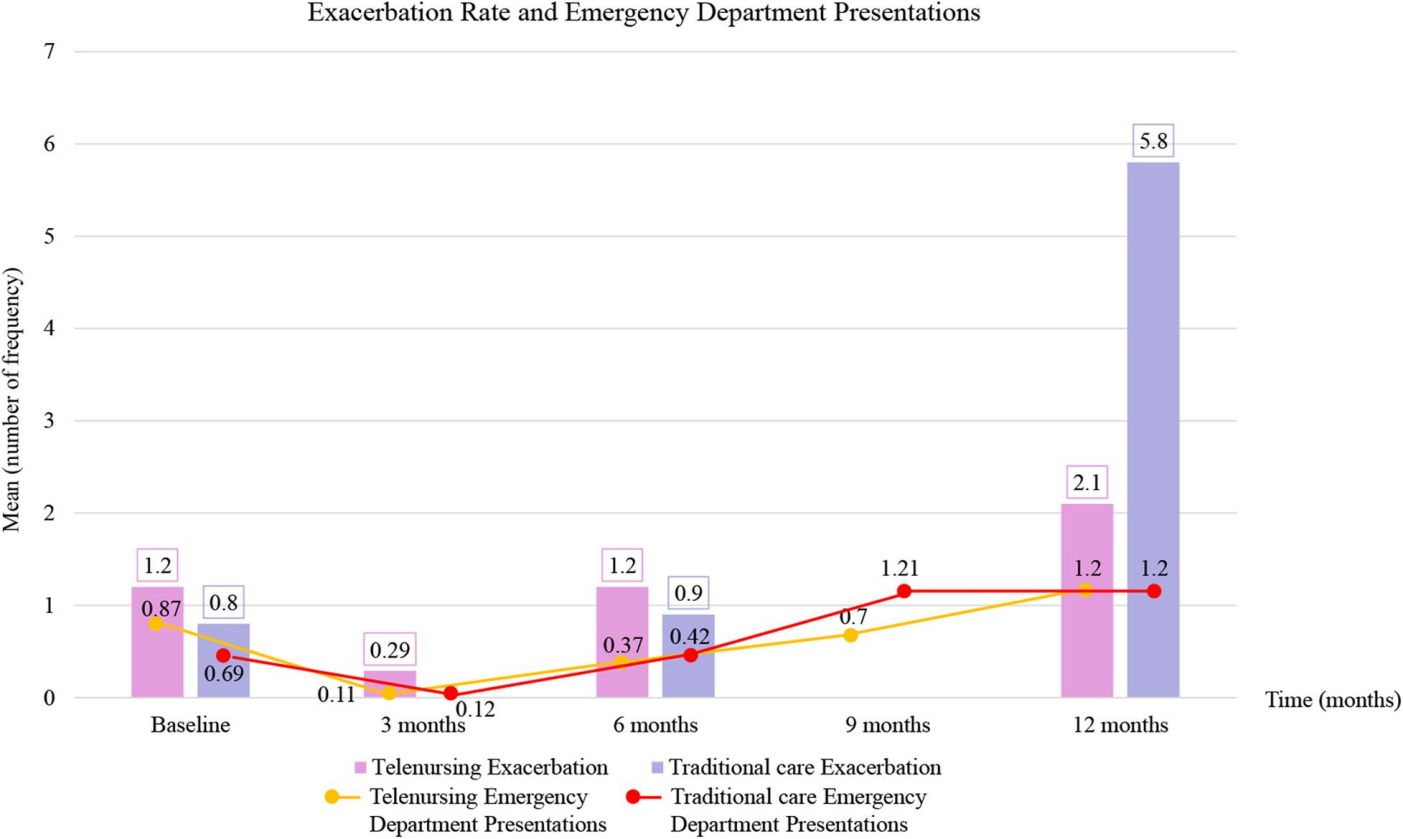
Exacerbation rates and emergency department presentations in the telenursing and traditional care groups.

Figure 4 displayed the relation over time between the exacerbation rates and ED presentations, comparing the telenursing group with the traditional care group.

Finally, hospital length of stay (LOS) remained largely unchanged between groups. In the control group, the mean LOS was 5.7 days (*n* *=* 272) at six months and 9.2 days (*n* *=* 172) at twelve months, compared with 5.2 days (*n* *=* 398) and 9.1 days (*n* *=* 337) in the intervention group, respectively.

These findings indicate that while telenursing interventions reduced the frequency of exacerbations and hospital admissions, they did not significantly shorten hospitalization duration once patients required inpatient care. This suggests that telenursing primarily acts on early detection and prevention of clinical deterioration, reducing hospitalization rates, but may have limited influence on the clinical course and management once hospitalization becomes necessary, as also observed by Sul et al. (2020) (87), who showed no difference in LOS, exclusively investigating the effectiveness of telemonitoring in COPD. However, the lack of consistent effects across all outcomes, particularly regarding emergency department visits and hospital length of stay, indicates that the overall impact of telenursing remains partial and should be interpreted with caution.

**Figure 5:**
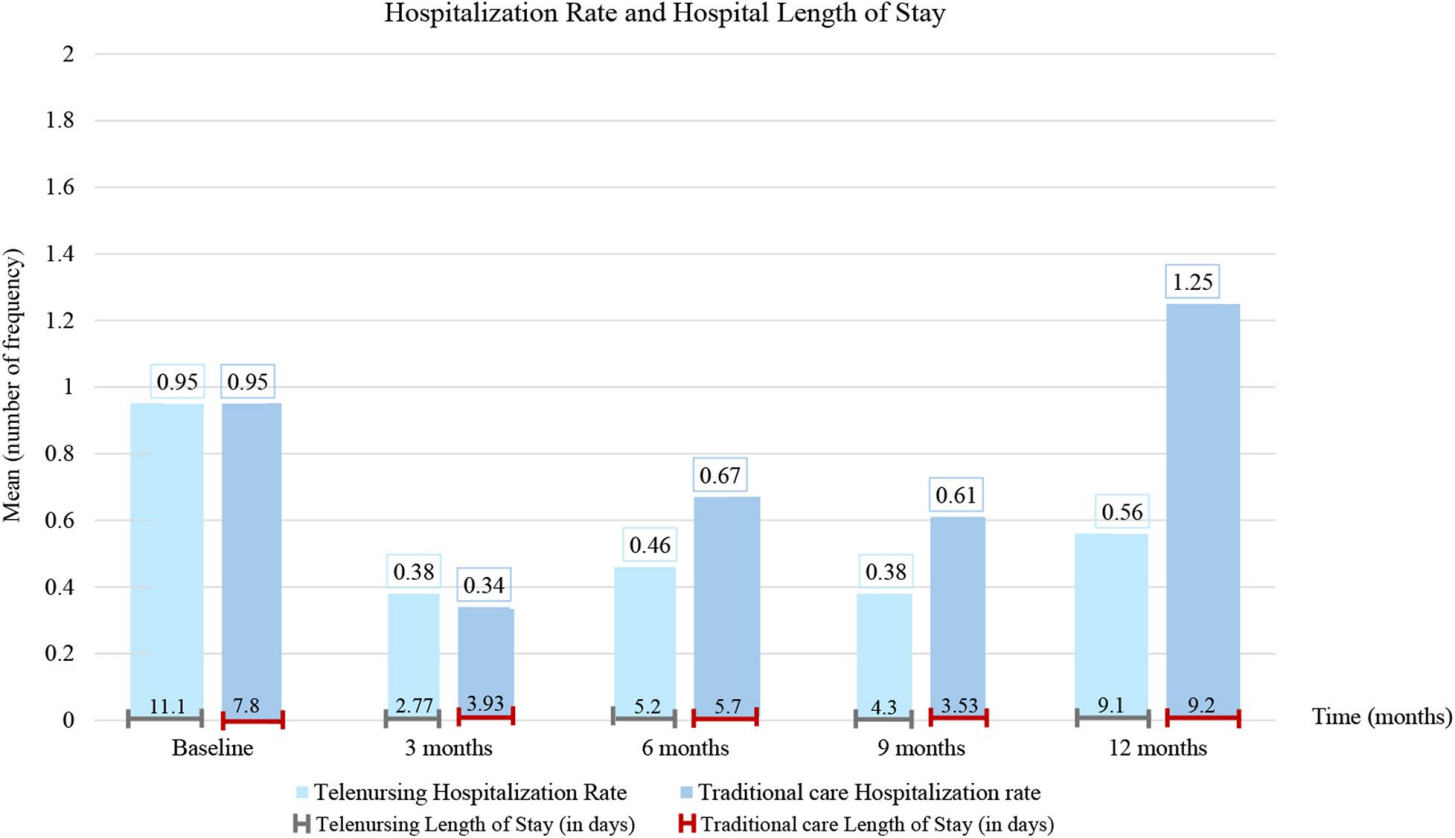
Hospitalization rate and hospital length of stay in the telenursing and traditional care groups.

Figure 5 displayed the relation over time between the exacerbation rates and ED presentations, comparing the telenursing group with the traditional care group.

Concerning treatment adherence, the included studies on telenursing interventions demonstrated high adherence, even if measured with widely different tools, both objective and self-reported. Adherence rates ranging from 40% to 97%, depending on the technology used and monitored parameter, only a minority of studies provided detailed quantitative results. For example, one study reported median adherence of 80% for daily physiological measurements (83% for blood pressure, 81% for oxygen saturation) (48), while another noted an increase in adherence from 45.6% to 85.3% over time (35). In contrast, studies in the control (traditional care) group showed minimal change in adherence, such as MARS scores increasing from 23.6 to 23.7 over 12 months (51) or self-reported adherence moving from 6.19 to 6.37 (46).

Regarding satisfaction and acceptability, intervention group satisfaction ranged from 77% to 94%, with mean satisfaction scores above 6/7 on the USE-Q in one study (34), and acceptability rates of 93.9% and 95.7% in two studies (35, 48). These findings are consistent with prior reviews of telemonitoring focusing exclusively on COPD patients, indicating generally high satisfaction and usability among patients with chronic respiratory disease (88, 89). However, in the remaining studies that used non-standardized tools to assess satisfaction and acceptability, the widespread use of unvalidated satisfaction scales has likely led to an overestimation of the intervention's benefits. Indeed, such non-standardized tools often fail to recognize the barriers to technology adoption, potentially masking technology fatigue, also called “Zoom fatigue”, behind high but superficial satisfaction scores (90).

However, the heterogeneity of measurement tools used in the studies included in the present systematic review, the limited number of studies reporting these outcomes, and short follow-up durations constrain definitive conclusions about patient-reported outcomes. Moreover, many studies relied on non-standardized tools to objectively assess the patient-reported outcomes, while only a minority of studies employed validated scales, limiting the comparability and robustness of these findings.

Furthermore, the notably higher adherence and satisfaction in telenursing groups may reflect greater patient engagement or novelty effects rather than sustained behavioral change over time (91). The novelty effect influences the way in which subjects react to the novelty of an innovation, which differs from the traditional way (85). However, the novelty effect tends to decrease over time; in particular, previous studies highlighted that the novelty effect lasts about 3 months (85). As a consequence, a novelty intervention that in the short term recorded improved patient-reported outcomes may encounter a decline in these outcomes over time (85).

Future research should utilize validated, standardized instruments to assess adherence, satisfaction, and usability, and explore whether high engagement translates into long-term clinical benefit. In fact, high levels of adherence, satisfaction, and acceptability may enhance the long-term benefits of telenursing by promoting sustained patient engagement and consistent self-management behavior, as found for other pharmacological and non-pharmacological types of long-term treatment (92–94).

### 4.1 Limitations, strengths, and future prospects

Despite promising findings on the potential role of telenursing, substantial heterogeneity among the included studies must be acknowledged. Variations in study design, intervention duration (ranging from 2 weeks to 26 months), sample size, technological tools, and outcome measures hindered the possibility of a quantitative meta-analysis. Although some outcomes appeared similar across studies, inconsistencies in measurement methods and follow-up durations prevented robust quantitative synthesis or subgroup analyses. Therefore, a structured narrative synthesis was considered the most appropriate approach to ensure a transparent and clinically meaningful interpretation of the available evidence.

Moreover, differences in disease severity and in the role of nurses within telemedicine programs limited comparability.

Methodological limitations, such as small sample sizes and incomplete reporting, also contributed to potential bias, as shown by the assessment quality of included studies, which reported a total of twelve studies with a high risk of bias.

Most studies were conducted in Europe and Australia, with fewer in North America and Asia, which limits generalizability to diverse healthcare contexts.

Collectively, the high degree of clinical and methodological heterogeneity across the included studies has important implications for the interpretation of the findings and reduces the overall strength and consistency of the evidence.

As a result, the observed effects of telenursing should be interpreted with caution, as they may not be directly comparable across studies nor generalizable to all clinical contexts. While some outcomes, such as exacerbation rates and hospital admissions, showed relatively consistent trends, others, particularly emergency department visits and hospital length of stay, demonstrated more variable or inconclusive results.

Additionally, patient-reported outcomes were often assessed using heterogeneous and non-validated tools, with a predominance of non-standardized self-reported measures, increasing the risk of measurement bias and limiting the reliability and comparability of these findings.

Future research should aim to standardize intervention protocols, outcome measures, and follow-up duration to allow comparability and strengthen evidence synthesis. Moreover, the development of interoperable digital infrastructures and user-friendly platforms would facilitate large-scale implementation and integration of telenursing into existing healthcare systems. Finally, policy-level support, training programs for healthcare professionals, and structured reimbursement models will be essential to ensure the long-term sustainability of telenursing services within national healthcare frameworks.

Among the main strengths of this systematic review, it represents one of the most comprehensive and up-to-date syntheses on telenursing in chronic respiratory diseases, including 39 studies and 4,340 patients, and extending the focus beyond COPD to encompass asthma and other chronic respiratory conditions. The review followed PRISMA guidelines and was registered on PROSPERO, ensuring methodological rigor and transparency. Furthermore, unlike previous reviews, the present study provides a multidimensional perspective by integrating patient-reported outcomes such as adherence, satisfaction, and acceptability, offering a broader understanding of current knowledge and gaps.

Another strength is the newness of telenursing, which represents a new frontier for chronic respiratory disease management, as shown by the relative actuality of the studies included, with the oldest study dating back to 2007 and most of the remaining studies dating back to the last 10 years, despite no date restrictions being applied in the literature search. The clinical strengths of telenursing were represented by the potential role in continuity of care, fostering patient empowerment, and allowing timely clinical decision-making based on continuous monitoring (10). While, as demonstrated in the present study, regarding the burden of healthcare systems, telenursing potentially reduced exacerbation and hospitalization rates, but did not shorten LOS once patients required inpatient care, suggesting that telenursing acted on prevention/early detection of clinical exacerbations, but had limited influence on the clinical course once hospitalization became necessary.

## 5 Conclusions

The present systematic review provides an updated and comprehensive synthesis of current evidence on the impact of telenursing in adult patients with chronic respiratory diseases, including COPD and asthma, compared with traditional care.

Overall, telenursing interventions were associated with improvements in some clinical outcomes compared to traditional care, although findings were not consistent across all outcomes. In particular, the improvements were recorded in reducing respiratory exacerbations and hospital admissions, particularly over long-term follow-up periods. These findings suggest that telenursing could play a preventive role, allowing early identification of clinical deterioration and timely adjustment of therapy, thereby potentially unburdening the healthcare system.

In contrast, emergency department presentations and hospital LOS did not significantly differ between telenursing and traditional care groups, indicating that while telenursing can reduce the occurrence of acute events, it may have a limited influence once inpatient care becomes necessary.

Adherence to treatment, patient satisfaction, and acceptability were consistently high in the telenursing group, with adherence rates reaching up to 97% and satisfaction levels exceeding 90%. These results confirm that patients perceive telenursing as useful, acceptable, and easy to integrate into daily life. However, the findings on the patient-reported outcomes may be considered with caution, given the heterogeneity of methods used to assess these outcomes and the frequent use of non-standardized tools, which can have led to engagement-related biases. Such positive behavioral and attitudinal factors may amplify the long-term benefits of telenursing by promoting sustained engagement and adherence to disease self-management strategies.

From a clinical and public health perspective, the findings of this review reinforce telenursing as a potential current frontier for unburdening healthcare systems related to chronic respiratory disease management. However, given the substantial heterogeneity across studies, these findings should be interpreted with caution and may not be fully generalizable across different healthcare settings.

## Data Availability

The original contributions presented in the study are included in the article/Supplementary Material, further inquiries can be directed to the corresponding author/s.
