# Supplementary material for: Unburdening healthcare systems through telenursing in chronic respiratory disease management: a systematic review
Source: Front Digit Health. 2026 May 21;8:1746693. doi: 10.3389/fdgth.2026.1746693 (PMC13233699; doi:10.3389/fdgth.2026.1746693)
Supplement: Supplementary file 1 [file Table1.docx]

Supplementary Material

**Supplementary Table 1.** Advanced search strategy performed in the three databases: PubMed, Web of Science, and Scopus.

| **Database** | **Advanced search strategy** | **Filters** | **Date of the latest search** |
| --- | --- | --- | --- |
| PubMed | ("nurs*"[All Fields] OR "Nursing"[All Fields] OR "Nurse's Role"[All Fields] OR "nursing care"[All Fields] OR "Nurse-Led Interventions"[All Fields] OR "Nursing Practice"[All Fields] OR "Advanced Practice Nurse"[All Fields] OR "Clinical Nurse Specialist"[All Fields] OR "Community Health Nurse"[All Fields] OR "Primary Care Nurse"[All Fields] OR "Nursing Assessment"[All Fields] OR "Nursing Intervention"[All Fields] OR "Nursing Support"[All Fields] OR "Nursing Education"[All Fields]) AND ("Telenursing"[All Fields] OR "Telehealth nursing"[All Fields] OR "Telemonitoring"[All Fields] OR "Remote Monitoring"[All Fields] OR "Remote Patient Monitoring"[All Fields] OR "Telemedicine"[All Fields] OR "eHealth"[All Fields] OR "mHealth"[All Fields] OR "Digital Health"[All Fields] OR "Telecare"[All Fields] OR "Home Telehealth"[All Fields] OR "Virtual Care"[All Fields] OR "Health Information Technology"[All Fields]) AND ("Chronic Pulmonary Disease"[All Fields] OR "Chronic Lung Disease"[All Fields] OR "Chronic Respiratory Disease"[All Fields] OR "Chronic Obstructive Pulmonary Disease"[All Fields] OR "COPD"[All Fields] OR "Asthma"[All Fields] OR "Cystic Fibrosis"[All Fields] OR "Emphysema"[All Fields] OR "Pneumonia"[All Fields] OR "Bronchitis"[All Fields] OR "Pulmonary Fibrosis"[All Fields] OR "Interstitial Lung Disease"[All Fields] OR "Bronchiectasis"[All Fields]) AND ("Clinical Outcomes"[All Fields] OR "Exacerbations"[All Fields] OR "Hospitalizations"[All Fields] OR "Emergency Visits"[All Fields] OR "Quality of Life"[All Fields] OR "Lung Function Test"[All Fields] OR "Pulmonary Rehabilitation Outcomes"[All Fields] OR "Adherence"[All Fields] OR "Satisfaction"[All Fields] OR "Cost-effectiveness"[All Fields]) | None | 06 April 2025 |
| Scopus | TITLE-ABS-KEY ( ( "Nurs*" OR "Nursing" OR "Nurse's Role" OR "nursing care" OR "Nurse-Led Interventions" OR "Nursing Practice" OR "Advanced Practice Nurse" OR "Clinical Nurse Specialist" OR "Community Health Nurse" OR "Primary Care Nurse" OR "Nursing Assessment" OR "Nursing Intervention" OR "Nursing Support" OR "Nursing Education" ) AND ( "Telenursing" OR "Telehealth nursing" OR "Telemonitoring" OR "Remote Monitoring" OR "Remote Patient Monitoring" OR "Telemedicine" OR "eHealth" OR "mHealth" OR "Digital Health" OR "Telecare" OR "Home Telehealth" OR "Virtual Care" OR "Health Information Technology" ) AND ( "Chronic Pulmonary Disease" OR "Chronic Lung Disease" OR "Chronic Respiratory Disease" OR "Chronic Obstructive Pulmonary Disease" OR "COPD" OR "Asthma" OR "Cystic Fibrosis" OR "Emphysema" OR "Pneumonia" OR "Bronchitis" OR "Pulmonary Fibrosis" OR "Interstitial Lung Disease" OR "Bronchiectasis" ) AND ( "Clinical Outcomes" OR "Exacerbations" OR "Hospitalizations" OR "Emergency Visits" OR "Quality of Life" OR "Lung Function Test" OR "Pulmonary Rehabilitation Outcomes" OR "Adherence" OR "Satisfaction" OR "Cost-effectiveness" ) ) | None | 06 April 2025 |
| Web of Science | ((TS = title-abs-key) AND (((((((((((((((((TS = "Nurs*") OR (TS = "Nursing")) OR (TS = "Nurse's Role")) OR (TS = "nursing care")) OR (TS = "Nurse-Led Interventions")) OR (TS = "Nursing Practice")) OR (TS = "Advanced Practice Nurse")) OR (TS = "Clinical Nurse Specialist")) OR (TS = "Community Health Nurse")) OR (TS = "Primary Care Nurse")) OR (TS = "Nursing Assessment")) OR (TS = "Nursing Intervention")) OR (TS = "Nursing Support")) OR (TS = "Nursing Education")) AND (((((((((((((TS = "Telenursing") OR (TS = "Telehealth nursing")) OR (TS = "Telemonitoring")) OR (TS = "Remote Monitoring")) OR (TS = "Remote Patient Monitoring")) OR (TS = "Telemedicine")) OR (TS = "eHealth")) OR (TS = "mHealth")) OR (TS = "Digital Health")) OR (TS = "Telecare")) OR (TS = "Home Telehealth")) OR (TS = "Virtual Care")) OR (TS = "Health Information Technology"))) AND (((((((((((((TS = "Chronic Pulmonary Disease") OR (TS = "Chronic Lung Disease")) OR (TS = "Chronic Respiratory Disease")) OR (TS = "Chronic Obstructive Pulmonary Disease")) OR (TS = "COPD")) OR (TS = "Asthma")) OR (TS = "Cystic Fibrosis")) OR (TS = "Emphysema")) OR (TS = "Pneumonia")) OR (TS = "Bronchitis")) OR (TS = "Pulmonary Fibrosis")) OR (TS = "Interstitial Lung Disease")) OR (TS = "Bronchiectasis"))) AND ((((((((((TS = "Clinical Outcomes") OR (TS = "Exacerbations")) OR (TS = "Hospitalizations")) OR (TS = "Emergency Visits")) OR (TS = "Quality of Life")) OR (TS = "Lung Function Test")) OR (TS = "Pulmonary Rehabilitation Outcomes")) OR (TS = "Adherence")) OR (TS = "Satisfaction")) OR (TS = "Cost-effectiveness")))) | None | 06 April 2025 |
